# SIRT5-mediated desuccinylation of the porcine deltacoronavirus M protein drives pexophagy to enhance viral proliferation

**DOI:** 10.1371/journal.ppat.1013163

**Published:** 2025-05-09

**Authors:** Zhuang Li, Wenbing Tang, Yinan Lai, Chaoqun Chen, Puxian Fang, Yanrong Zhou, Liurong Fang, Shaobo Xiao

**Affiliations:** 1 National Key Laboratory of Agricultural Microbiology, College of Veterinary Medicine, Huazhong Agricultural University, Wuhan, China; 2 Key Laboratory of Preventive Veterinary Medicine in Hubei Province, Cooperative Innovation Center for Sustainable Pig Production, Wuhan, China; 3 College of Veterinary Medicine, Shandong Agricultural University, Tai’an, China; Washington University School of Medicine in Saint Louis: Washington University in St Louis School of Medicine, UNITED STATES OF AMERICA

## Abstract

Porcine deltacoronavirus (PDCoV) is an emerging enteropathogenic coronavirus capable of infecting various animal species, including humans. In this study, we explored the roles of sirtuins (SIRTs), a conserved family of protein deacylases and mono-adenosine diphosphate-ribosyltransferases, in PDCoV replication. Surprisingly, we found that SIRT5—a unique member of SIRTs with distinct desuccinylation, demalonylation, and deglutarylation activities—is a proviral factor essential for PDCoV replication; its catalytic activities are crucial in this process. Mechanistically, SIRT5 interacts with and desuccinylates the PDCoV membrane (M) protein. This modification activates the ataxia-telangiectasia mutated (ATM) pathway, facilitates ubiquitination of peroxisomal biogenesis protein 5 (PEX5), and recruits sequestosome 1 (SQSTM1/p62) to initiate selective peroxisomal autophagy (pexophagy). The pexophagy process disrupts peroxisomal function, elevates reactive oxygen species (ROS) levels, and suppresses type I and III interferon production, thereby enhancing viral replication. We also identified lysine 207 (K207) as the primary succinylation site of the M protein. Mutations mimicking the desuccinylated or succinylated states of K207 substantially influence viral replication and the ability to induce pexophagy. These findings reveal a novel role for SIRT5 in regulating pexophagy during viral infection and suggest a therapeutic target for efforts to combat coronavirus infections.

## Author summary

Porcine deltacoronavirus (PDCoV) poses significant risks to both animal health and human safety. In this study, we identified SIRT5 as a crucial proviral factor that enhances PDCoV replication. By mediating the desuccinylation of the viral M protein, SIRT5 activates pexophagy, which disrupts peroxisomal function, increases reactive oxygen species (ROS), and suppresses immune responses, thereby promoting viral replication. The identification of lysine 207 as a critical succinylation site on the M protein further underscores the importance of post-translational modifications in regulating PDCoV replication. These findings suggest that SIRT5 may serve as a promising target for therapeutic interventions against coronavirus infections.

## Introduction

Porcine deltacoronavirus (PDCoV) is an emerging enteropathogenic coronavirus that primarily affects suckling piglets, causing acute watery diarrhea, vomiting, dehydration, and death [1,2]. It was initially identified in Hong Kong in 2012 [3], and the first outbreak in pig farms was reported in the United States in 2014. Since then, PDCoV outbreaks and epidemics have been documented in multiple countries or regions, including South Korea, mainland China, Thailand, Japan, and Vietnam, resulting in substantial economic losses for the global swine industry [4–10]. A significant concern regarding PDCoV is its capacity for cross-species transmission. In addition to pigs, PDCoV can infect chickens, turkey poults, calves, and mice [1,11–15]. Notably, PDCoV has been detected in plasma samples from three Haitian children with acute undifferentiated febrile illness, highlighting its potential threat to human health [16]. As a representative member of the *Deltacoronavirus* genus and the only deltacoronavirus successfully isolated in a cell culture system, PDCoV has a genome of approximately 25.4 kb. Its genome encodes two large polyproteins, four structural proteins (spike [S], envelope [E], membrane [M], and nucleocapsid [N]), and three accessory proteins [17,18]. The M protein is a major structural protein and the most abundant component of the viral envelope [19]; it is highly conserved among coronaviruses [20], where it plays key roles in viral assembly, budding, and immune evasion [21–23].

Sirtuins (SIRTs) are a conserved family of protein deacylases and mono-adenosine diphosphate-ribosyltransferases found in organisms ranging from bacteria to humans. In mammals, seven SIRT isoforms (SIRT1–7) have been identified. These isoforms share highly conserved nicotinamide adenine dinucleotide (NAD)-binding domains and catalytic sites; differences in N- and C-terminal regions determine their subcellular localization and substrate specificity [24,25]. SIRT1, SIRT6, and SIRT7 are primarily located in the nucleus; SIRT7 exhibits particular enrichment in the nucleolus [26–28]. SIRT2 is mainly cytoplasmic but translocates to the nucleus during mitosis [26,29]. SIRT3, SIRT4, and SIRT5 are predominantly localized in mitochondria, where they regulate energy metabolism, redox homeostasis, and aging-related processes [30–32]. Although SIRT5 is categorized as a protein deacetylase, its deacetylase activity is weak relative to that of other SIRTs. Instead, SIRT5 exhibits a strong affinity for negatively charged acyl-lysine modifications and primarily mediates desuccinylation, demalonylation, and deglutarylation [33,34]. Furthermore, SIRT5 has emerged as a critical regulator of autophagy under various physiological and pathological conditions. Previous studies have demonstrated that SIRT5 not only enhances autophagy but also acts as a proliferative factor in several cancers, including colorectal, gastric, and breast cancers, as well as osteosarcoma [35–38]. A recent study demonstrated that SIRT5 induces autophagy via desuccinylation of myb1 membrane trafficking protein, thereby mitigating myocardial infarction [39]. These findings position SIRT5 as a key regulator of autophagy with potential therapeutic implications in cancer progression and cardiac protection. Emerging evidence also suggests that SIRT5 plays a critical role in severe acute respiratory syndrome coronavirus 2 (SARS-CoV-2) infection by interacting with viral nonstructural protein 14 (nsp14). The succinylation of SIRT5 appears to be important for this interaction [40,41]. Although primarily characterized as a mitochondrial protein, SIRT5 is also present in the cytosol, nucleus, and peroxisomes [42,43].

Peroxisomes are highly dynamic organelles central to essential biological processes such as fatty acid β-oxidation, lipid biosynthesis, and reactive oxygen species (ROS) metabolism. In addition to these metabolic functions, peroxisomes serve as antiviral signaling platforms, contributing to innate immune activation [44,45]. The biogenesis and degradation of peroxisomes are tightly regulated to maintain cellular homeostasis. Peroxisomal biogenesis proteins (PEXs) are responsible for the assembly of peroxisomes and regulation of their abundance [46,47]. Peroxisome degradation occurs through pexophagy, a selective form of autophagy comprising peroxisome sequestration within autophagosomes and subsequent degradation in autolysosomes [48]. However, there is uncertainty regarding whether SIRT5 contributes to the regulation of pexophagy, particularly in the context of viral infections.

In this study, we screened all seven SIRTs and identified SIRT5 as a proviral factor for PDCoV proliferation. Further investigation revealed that SIRT5 interacts with the PDCoV M protein and desuccinylates it at lysine 207 (K207), activating pexophagy via the ataxia-telangiectasia mutated (ATM)-PEX5-p62 axis. This activation leads to peroxisome dysfunction, which subsequently enhances viral proliferation. The discovery of this novel mechanism provides valuable insights for developing therapeutic strategies against PDCoV and other pathogenic coronaviruses.

## Results

### Screening SIRTs that regulate PDCoV replication

**Fig 1:**
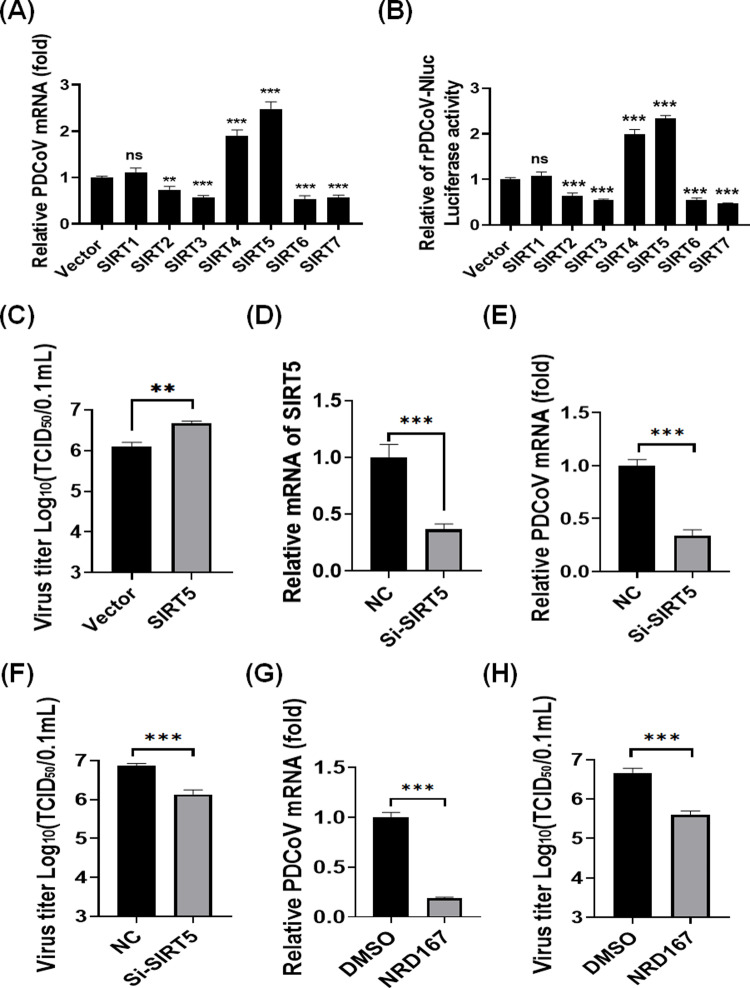
Screening SIRTs that regulate PDCoV replication. **(A-B)** LLC-PK1 cells were transfected with individual SIRT expression constructs (SIRT1–SIRT7) and subsequently infected with PDCoV (A) or a PDCoV reporter virus expressing nanoluciferase (PDCoV-Nluc) (**B**). Cell samples were harvested and analyzed by quantitative reverse transcription polymerase chain reaction (qRT-PCR) (A) and luciferase assays (**B**). (**C**) LLC-PK1 cells were transfected with pCAGGS-Flag-SIRT5 or an empty vector for 12 h, then infected with PDCoV (multiplicity of infection [MOI] = 0.5). At 12 hpi, cells were collected and subjected to TCID_50_ assays. (**D-F**) LLC-PK1 cells were transfected with SIRT5-specific siRNA or control siRNA, then infected with PDCoV (MOI = 0.5) for 12 h. Cells were harvested for qRT-PCR to measure relative SIRT5 mRNA levels (**D**), viral mRNA levels (**E**), and TCID_50_ assays to determine viral titers (**F**). **(G-H**) LLC-PK1 cells were pretreated with NRD167 (10 µM) for 2 h, then infected with PDCoV (MOI = 0.5). At 12 hpi, cells were collected and analyzed by qRT-PCR for viral RNA levels (**G**) and TCID_50_ assays for viral titers (**H**). **p < 0.01, ***p < 0.001. ns, not significant.

To investigate the roles of SIRTs in PDCoV infection, we overexpressed individual SIRTs (SIRT1–7) in LLC-PK1 cells and infected the cells with PDCoV. We found that overexpression of SIRT2, SIRT3, SIRT6, and SIRT7 significantly inhibited PDCoV replication, whereas SIRT4 and SIRT5 significantly enhanced replication, as measured by quantitative reverse transcription polymerase chain reaction (qRT-PCR) (Figs 1A and S1A). Next, we used a PDCoV reporter virus expressing nanoluciferase (Nluc) to evaluate the impact of each SIRT on viral replication. The findings were consistent with the results of qRT-PCR (Fig 1B). Previous studies have shown that SIRT5 is involved in SARS-CoV-2 infection [40,49]. Moreover, SIRT5 possesses distinct enzymatic functions—desuccinylation, demalonylation, and deglutarylation—and its unexpected role in enhancing PDCoV replication. Taken these into account, we selected SIRT5 for further investigation.

To further investigate the role of SIRT5 in PDCoV proliferation, we conducted 50% tissue culture infectious dose (TCID_50_) assays. Overexpression of SIRT5 significantly increased viral titers (Fig 1C). To complement these findings, we used SIRT5-specific small interfering RNA (Si-SIRT5) to knock down SIRT5 expression in LLC-PK1 cells (Figs 1D and S1B). Compared with negative control siRNA (NC), Si-SIRT5 significantly reduced PDCoV replication (Fig 1E and 1F). Additionally, we examined the effect of NRD167, a specific SIRT5 inhibitor [50], on PDCoV replication in LLC-PK1 cells. Treatment with NRD167 led to a substantial decrease in viral RNA levels (Fig 1G) and viral titers (Fig 1H), confirming that SIRT5 enhances PDCoV proliferation.

### SIRT5 interacts with the PDCoV M protein

**Fig 2:**
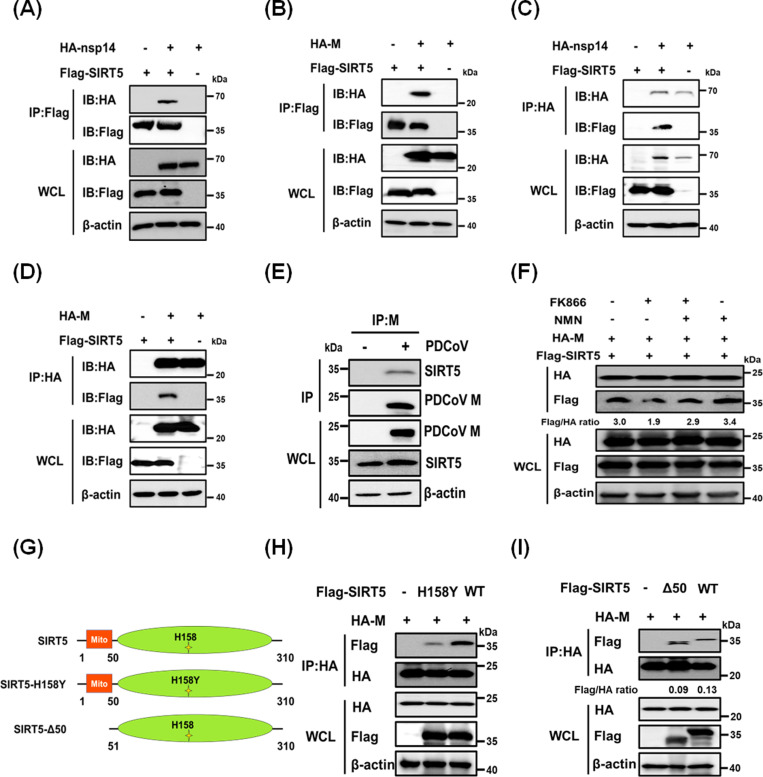
SIRT5 interacts with the PDCoV M protein. (**A-D**) HEK-293T cells were co-transfected with pCAGGS-Flag-SIRT5 and pCAGGS-HA-nsp14 or pCAGGS-HA-M for 24 h. The cells were lysed and subjected to immunoprecipitation using anti-Flag antibody (A, B) or anti-HA antibody (**C**, **D**). (**E**) LLC-PK1 cells were infected with PDCoV (MOI = 0.5) for 12 h. The cells were lysed, and co-IP assays were performed using anti-M antibody. Whole-cell lysate (WCL) and IP complexes were analyzed by western blotting with antibodies against Flag, HA, PDCoV M, SIRT5, or β-actin. (**F**) HEK-293T cells were co-transfected with pCAGGS-HA-M and pCAGGS-Flag-SIRT5, followed by treatment with FK866 (20 nM), FK866 combined with NMN (100 µM), or NMN alone. Cells were lysed and analyzed by western blotting. Ratios below the images represent the protein levels of Flag relative to HA, as quantified by the ImageJ software. (**G**) Schematic representation of SIRT5 mutants. (**H-I**) HEK-293T cells were co-transfected with pCAGGS-HA-M and either pCAGGS-Flag-SIRT5, Flag-SIRT5-H158Y (**H**), or Flag-SIRT5-Δ50 (I) for 24 h. The cells were lysed, and immunoprecipitation was performed using anti-HA antibody. WCL and IP complexes were analyzed by western blotting. Ratios below the images represent the protein levels of Flag relative to HA, as quantified by the ImageJ software.

To examine the mechanism by which SIRT5 enhances PDCoV replication, we screened for PDCoV-encoded proteins that interact with SIRT5. HEK-293T cells were co-transfected with pCAGGS-Flag-SIRT5 and hemagglutinin (HA)-tagged plasmids encoding individual PDCoV proteins. Co-immunoprecipitation (co-IP) assays using anti-Flag antibodies revealed that SIRT5 interacts with nsp2, nsp10, nsp14, and the M protein (Figs 2A, 2B and S2A). To corroborate these interactions, we performed reverse co-IP assays using anti-HA antibodies. These experiments confirmed robust interactions between SIRT5 and both nsp14 and the M protein; interactions with nsp2 and nsp10 were not observed (Figs 2C, 2D and S2B). Combining the findings from both forward and reverse co-IP experiments, we concluded that SIRT5 interacts with PDCoV nsp14 and M protein in an overexpression system. Considering previous reports that SIRT5 interacts with SARS-CoV-2 nsp14 but does not directly modify it [40,49], we focused on the interaction between SIRT5 and the PDCoV M protein. To validate this interaction in the context of PDCoV infection, we conducted co-IP experiments using an anti-M protein antibody. The results confirmed that M protein interacts with endogenous SIRT5 in PDCoV-infected cells (Fig 2E).

SIRT5 functions as a NAD ⁺ -dependent histone deacetylase, requiring NAD⁺ as a co-substrate for its enzymatic activity [51]. To determine whether the interaction between SIRT5 and M protein depends on the enzymatic activity of SIRT5, we first assessed the impact of intracellular NAD⁺ levels on their binding. FK866, an inhibitor of nicotinamide phosphoribosyltransferase (the rate-limiting enzyme in the NAD⁺ salvage pathway [52]), was utilized to deplete cellular NAD⁺ levels; the NAD⁺ precursor nicotinamide mononucleotide (NMN) was used to restore NAD⁺ levels [53]. As shown in Fig 2F, FK866 reduced the interaction between SIRT5 and M protein, whereas NMN enhanced this interaction. Furthermore, the reduced interaction caused by FK866 was rescued by NMN, suggesting that the interaction between SIRT5 and M protein is dependent on the enzymatic activity of SIRT5. To explore this possibility, we tested whether SIRT5 interacts with an enzyme-deficient mutant of SIRT5 (H158Y) [54] (Fig 2G). The results showed that the binding of SIRT5-H158Y to M protein was substantially reduced compared with wild-type SIRT5 (Fig 2H), confirming that the catalytic activity of SIRT5 is essential for its interaction with the M protein.

### The PDCoV M protein and SIRT5 co-localize to peroxisomes

**Fig 3:**
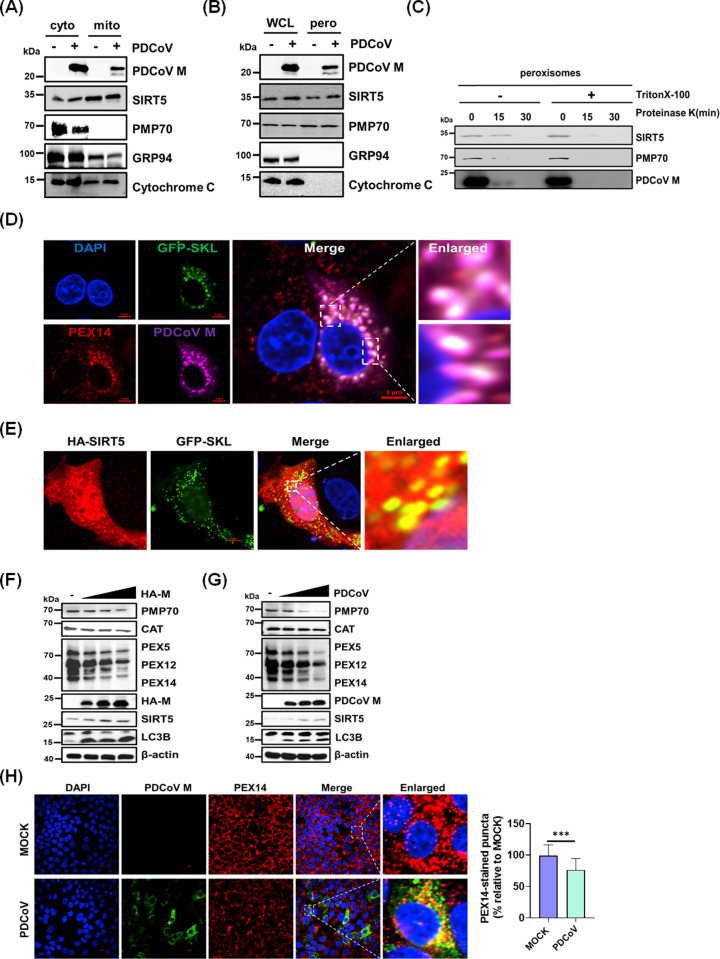
The PDCoV M protein and SIRT5 co-localize to peroxisomes and induce peroxisome degradation. (**A-B**) Mitochondria (A) and peroxisomes (B) were isolated from PDCoV-infected or mock-infected LLC-PK1 cells and analyzed by western blotting using the indicated antibodies. (**C**) Peroxisomes isolated from LLC-PK1 cells were treated with proteinase K (100 μg/mL) with or without pre-treatment with 1% Triton X-100. Samples were collected at the indicated time points and analyzed by western blotting. (**D**) LLC-PK1 cells were transfected with GFP-SKL and infected with PDCoV. Cells were fixed and subjected to immunofluorescence assay (IFA) using anti-PEX14 or anti-M antibodies. Nuclei were counterstained with 4′,6-diamidino-2-phenylindole (DAPI). Scale bar, 5 µm. **(E)** LLC-PK1 cells were co-transfected with GFP-SKL and HA-SIRT5 for 24 h. Cells were fixed and analyzed by IFA using anti-HA antibody. Nuclei were counterstained with DAPI. Scale bar, 5 µm. (**F-G**) LLC-PK1 cells were transfected with pCAGGS-HA-M (F) or infected with PDCoV at increasing doses (**G**), then collected for western blotting analysis. (**H**) LLC-PK1 cells were mock-infected or infected with PDCoV. Cells were fixed and analyzed by IFA using anti-PEX14 or anti-M antibodies. Nuclei were counterstained with DAPI. Scale bar, 10 µm. The percentage of PEX14-stained puncta in infected cells compared with the control group was quantified.

To better understand the interaction between SIRT5 and the PDCoV M protein, we analyzed their subcellular localization in a co-expression system. We used an N-terminal deletion mutant of SIRT5 (SIRT5-Δ50), which cannot localize to mitochondria (Fig 2G). Despite this deletion, SIRT5-Δ50 still interacted with the M protein (Fig 2I), indicating that the interaction is not solely dependent on the mitochondrial localization of SIRT5. This finding prompted us to investigate other potential sites of co-localization. A previous study showed that SIRT5 can localize to both peroxisomes and mitochondria [43]. Therefore, we hypothesized that M protein may also localize to peroxisomes. To test this hypothesis, we conducted cell fractionation experiments in PDCoV-infected LLC-PK1 cells. The results confirmed the presence of the peroxisomal membrane protein PMP70 in the peroxisomal fraction, whereas cytochrome C was exclusively detected in the mitochondrial fraction (Fig 3A and 3B). The absence of GRP94 in the peroxisomal fraction validated its purity and ruled out contamination from the endoplasmic reticulum. Importantly, endogenous SIRT5 and the PDCoV M protein were detected in both peroxisomal and mitochondrial fractions (Fig 3A and 3B), supporting the hypothesis that M protein localizes to these two compartments. In addition, we found that the PDCoV M protein interacts with peroxisomal proteins PEX5, PEX12, and PEX14, suggesting these interactions may facilitate its localization to peroxisomes (S3 Fig). Next, we performed proteinase K treatment and western blotting analysis on purified peroxisomes after permeabilization with Triton X-100. The results showed that proteinase K rapidly degraded PMP70 and M protein within 15 min; the degradation rate of M protein accelerated after Triton X-100 permeabilization (Fig 3C). These results collectively demonstrate that the M protein is present in peroxisomes, where it localizes to peroxisomal membranes.

To confirm the peroxisomal localization of the PDCoV M protein, we used an additional peroxisomal marker, green fluorescent protein (GFP)-Ser-Lys-Leu (GFP-SKL). This marker contains the peroxisomal matrix-targeting signal peptide SKL fused to the C-terminus of GFP, ensuring localization to peroxisomes [55]. We validated the localization of GFP-SKL to peroxisomes via co-localization with the peroxisome-associated protein PEX14 (S4 Fig). As shown in Fig 3D and 3E, the PDCoV M protein and SIRT5 co-localized with GFP-SKL, providing further evidence that the PDCoV M protein localizes to peroxisomes.

### The PDCoV M protein induces peroxisome degradation via pexophagy

Considering the peroxisomal localization of the PDCoV M protein, we investigated whether the M protein influences the expression of peroxisome-associated proteins. Accordingly, HEK-293T cells were transfected with an HA-tagged M protein expression plasmid (pCAGGS-HA-M), then subjected to western blotting analysis. The results showed that M protein caused dose-dependent decreases in the expression levels of peroxisome-associated proteins, including PMP70, PEX5, PEX12, PEX14, and catalase (CAT) (Fig 3F). Similarly, PDCoV infection led to decreased expression of these peroxisome-associated proteins (Fig 3G) and a reduction in the number of peroxisomes, as indicated by endogenous PEX14 staining (Fig 3H). These findings suggest that both PDCoV infection and overexpression of the M protein induce peroxisome degradation.

**Fig 4:**
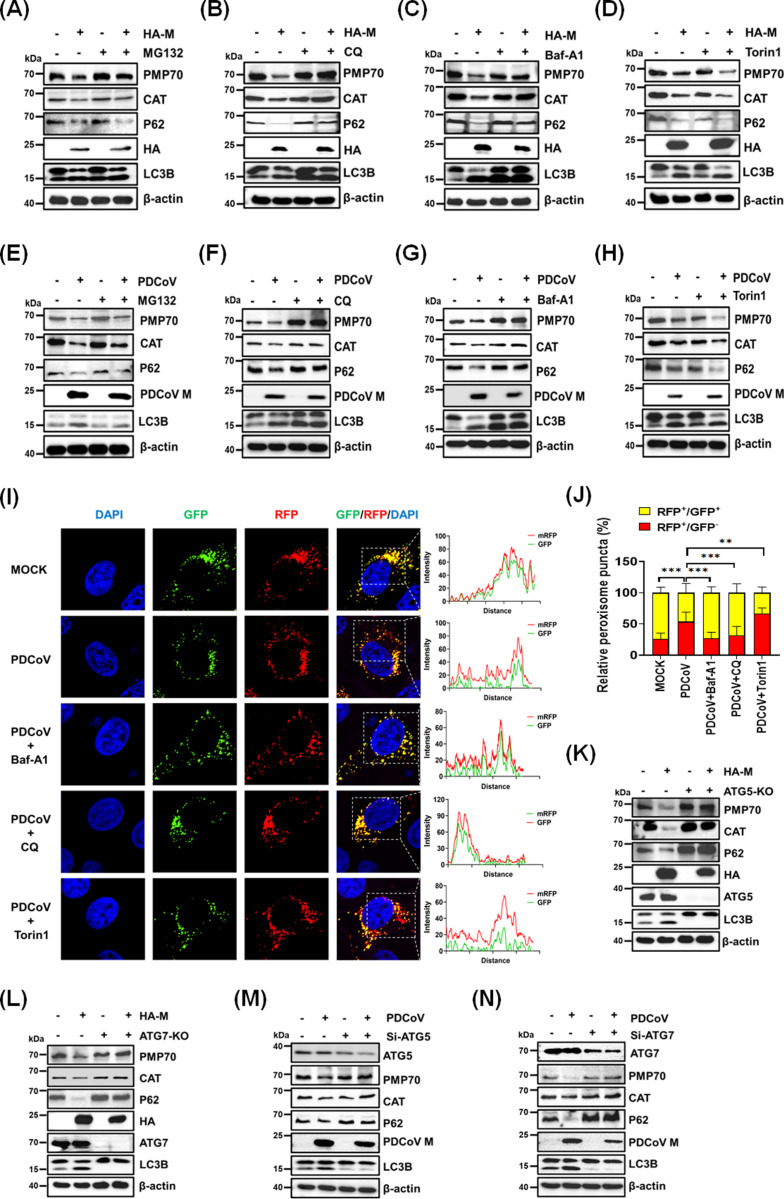
The PDCoV M protein induces pexophagy. **(A-D)** HEK-293T cells were transfected with pCAGGS-HA-M and treated with (A) MG132 (10 µM), (B) CQ (10 µM), (C) Baf-A1 (50 nM), or (D) Torin1 (1 µM) for 12 h. Cell lysates were collected and analyzed by western blotting. (E-H) LLC-PK1 cells were infected with PDCoV and treated with (E) MG132 (10 µM), (F) CQ (10 µM), (G) Baf-A1 (50 nM), or (H) Torin1 (1 µM) for 12 h. Cell lysates were collected and subjected to western blotting analysis. (I-J) LLC-PK1 cells expressing GFP-RFP-SKL were infected with PDCoV and treated with DMSO, Baf-A1 (50 nM), CQ (10 µM), or Torin1 (1 µM). Cells were fixed and examined by microscopy to assess the colocalization of RFP and GFP signals (I). The numbers of RFP and GFP double-positive peroxisomal puncta (RFP^+^/GFP^+^) and RFP-positive/GFP-negative peroxisomal puncta (RFP^+^/GFP^-^) were quantified using ImageJ software (J). (K-L) ATG5-KO (K), ATG7-KO (L), and wild-type HEK-293T cells were transfected with pCAGGS-HA-M for 24 h. Cell lysates were collected and analyzed by western blotting with the indicated antibodies. (M-N) LLC-PK1 cells were transfected with siRNAs targeting ATG5 (M) or ATG7 (N), then infected with PDCoV (MOI = 0.5) for 12 h. Cells were harvested, and lysates were analyzed by western blotting with the indicated antibodies.

To identify the pathway through which the M protein and PDCoV degrade peroxisomes, we conducted experiments involving HA-M overexpression or PDCoV infection, with or without the proteasome inhibitor MG132 and the lysosome inhibitors chloroquine (CQ) and bafilomycin A1 (Baf-A1). The results demonstrated that the degradation of PMP70 and CAT was unaffected by MG132 but was inhibited by CQ and Baf-A1 (Fig 4A–C, 4E–G), indicating that M protein overexpression and PDCoV infection mediate peroxisome degradation through autophagy. Additionally, treatment with the mechanistic target of rapamycin inhibitor Torin1, a known inducer of autophagy, enhanced M protein-induced autophagy and promoted the degradation of PMP70 and CAT (Fig 4D). Similar results were observed in PDCoV-infected cells (Fig 4H). These findings suggest that peroxisome degradation occurs via pexophagy under conditions of M protein overexpression and PDCoV infection.

To visualize pexophagy, we utilized a peroxisome-targeted red fluorescent protein (RFP)-GFP-SKL probe. In PDCoV-infected cells, the GFP signal was quenched in acidic lysosomes, resulting in fewer correlated RFP/GFP signals (Fig 4I); these results implied the occurrence of pexophagy. Cells treated with Baf-A1 or CQ displayed RFP/GFP double-positive puncta, whereas Torin1 treatment led to an increase in RFP single-positive puncta. These results suggested that PDCoV infection increases the colocalization of peroxisomes and lysosomes, confirming the occurrence of pexophagy (Fig 4I and 4J). Finally, we investigated the role of autophagy-related genes in pexophagy using ATG5 and ATG7 knockout (ATG5-KO, ATG7-KO) and wild-type HEK-293T cells. In wild-type cells, M protein reduced the levels of peroxisomal markers PMP70 and CAT. However, these reductions were absent in ATG5-KO and ATG7-KO cells (Fig 4K and 4L). Similarly, knockdown of ATG5 or ATG7 using specific siRNA abolished the inhibitory effects of PDCoV infection on these peroxisomal markers (Fig 4M and 4N). These results demonstrate that PDCoV infection and M protein overexpression can induce pexophagy.

### The PDCoV M protein induces pexophagy through SIRT5

**Fig 5:**
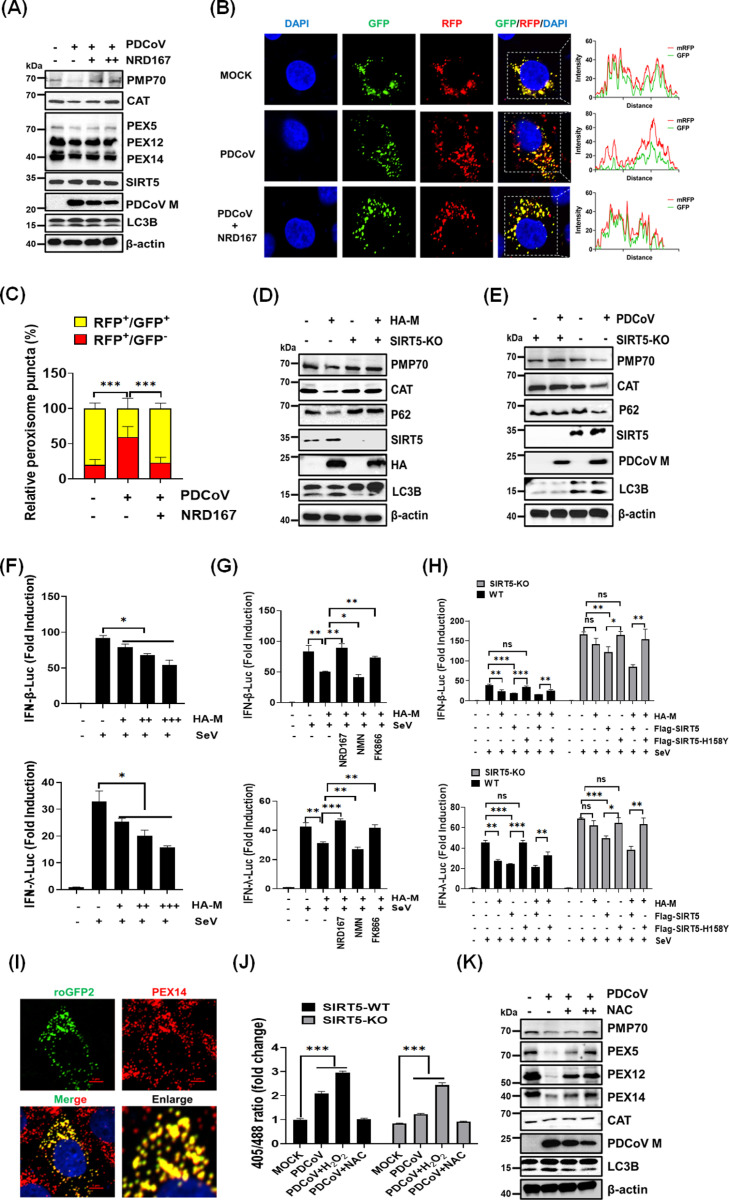
The PDCoV M protein triggers SIRT5-dependent pexophagy and impairs peroxisomal function. (**A**) LLC-PK1 cells were treated with NRD167 (10, 20 µM) or left untreated, then infected with PDCoV. The protein levels of peroxisomal markers were analyzed by western blotting using the indicated antibodies. (**B-C**) LLC-PK1 cells expressing GFP-RFP-SKL were infected with PDCoV and treated with either DMSO or NRD167 (10 µM). Cells were fixed and analyzed by microscopy to evaluate the colocalization of RFP and GFP signals (**B**). The numbers of RFP^+^/GFP^+^ and RFP^+^/GFP^-^ peroxisomal puncta were quantified using ImageJ software (**C**). (**D**) SIRT5-KO and wild-type HEK-293T cells were transfected with the pCAGGS-HA-M plasmid. Cell lysates were collected and analyzed by western blotting with the indicated antibodies. (**E**) Wild-type and SIRT5-KO LLC-PK1 cells were infected with PDCoV. Cell lysates were collected and subjected to western blotting analysis using the indicated antibodies. (**F-G**) HEK-293T cells were co-transfected with IFN-β-Luc or IFN-λ-Luc and pRL-TK plasmids for 24 h, along with increasing amounts of pCAGGS-HA-M or an empty vector (**F**), or treated with the indicated inhibitors (**G**), then infected with Sendai virus (SeV) for 12 h. Cells were lysed and analyzed using dual-luciferase assays. (**H**) SIRT5-KO and wild-type HEK-293T cells were co-transfected with IFN-β-Luc or IFN-λ-Luc and pRL-TK plasmids for 24 h, along with pCAGGS-HA-M, pCAGGS-Flag-SIRT5, pCAGGS-Flag-SIRT5-H158Y, or an empty vector. Cells were then infected with SeV for 12 h, lysed, and subjected to dual-luciferase assays. (**I-J**) LLC-PK1 cells were transfected with roGFP2 plasmids to monitor peroxisomal ROS levels. Fixed cells were analyzed by IFA using anti-PEX14 antibody; nuclei were counterstained with DAPI (**I**). Oxidative stress levels in peroxisomes were quantified by measuring the 405/488 nm excitation ratio, as described in the Materials and Methods (**J**). (**K**) LLC-PK1 cells were treated with NAC or left untreated, then infected with PDCoV. The expression levels of peroxisomal markers were analyzed via western blotting using the indicated antibodies. ns, not significant, *p < 0.05, **p < 0.01, and ***p < 0.001.

Considering that SIRT5 interacts with the M protein, which induces pexophagy, we investigated whether SIRT5 contributes to PDCoV-induced pexophagy. We treated cells with the SIRT5 inhibitor NRD167 during PDCoV infection. The results showed that NRD167 treatment strongly inhibited the PDCoV-induced autophagic degradation of peroxisomal proteins PMP70, PEX5, PEX12, and PEX14 (Fig 5A). Additionally, in cells expressing the peroxisome-targeted RFP-GFP-SKL probe, NRD167 treatment increased the number of peroxisomal puncta exhibiting both RFP and GFP signals. This observation indicated a reduction in pexophagy levels. These findings suggest that a SIRT5 inhibitor can suppress PDCoV-induced pexophagy (Fig 5B and 5C).

To confirm the role of SIRT5 in mediating pexophagy induced by PDCoV and its M protein, we generated SIRT5 knockout (SIRT5-KO) HEK-293T and LLC-PK1 cell lines (S5 Fig). In wild-type HEK-293T cells, M protein overexpression increased autophagic flux, as evidenced by p62 degradation and elevated LC3-II levels. However, in SIRT5-KO HEK-293T cells, M protein overexpression decreased autophagic flux. The degradation of PMP70 and CAT, observed in wild-type cells during M protein overexpression, was absent in SIRT5-KO HEK-293T cells (Fig 5D). Similarly, in SIRT5-KO LLC-PK1 cells infected with PDCoV, the degradation of PMP70 and CAT was suppressed relative to wild-type LLC-PK1 cells, indicating that peroxisome degradation was impaired (Fig 5E). These results demonstrate that PDCoV M protein-induced pexophagy requires the presence and enzymatic activity of SIRT5, revealing a potential mechanism in which SIRT5 acts as a critical regulator of pexophagy during PDCoV infection.

### The PDCoV M protein disrupts peroxisome function via pexophagy

Peroxisomes are critical for innate immunity and cellular metabolism, particularly in antiviral responses and ROS production [45,56]. Considering that the M protein induces pexophagy, we hypothesized that it disrupts peroxisomal functions, thereby impairing ROS production and antiviral immunity. To test this hypothesis, we first assessed the impact of M protein on interferon (IFN) production. The results showed that M protein overexpression inhibited the production of IFN-β and IFN-λ in a dose-dependent manner during Sendai virus infection (Fig 5F). Next, we evaluated the role of SIRT5 in this process by treating M protein-expressing cells with a SIRT5 inhibitor (NRD167), an NAD⁺ agonist (NMN), or an NAD⁺ synthesis inhibitor (FK866). We found that NRD167 and FK866 restored IFN production, but NMN enhanced the inhibitory effects of the M protein on IFN production (Fig 5G), demonstrating the involvement of SIRT5 in this inhibition. To further validate the role of SIRT5, we expressed either wild-type SIRT5 or an enzymatically inactive mutant (SIRT5-H158Y) in both wild-type and SIRT5-KO cells. In wild-type cells, only active SIRT5 suppressed IFN-β and IFN-λ production, whereas the inactive mutant had no effect. In SIRT5-KO cells, the M protein’s inhibitory effect on IFN production was absent unless active SIRT5 was reintroduced (Fig 5H). These findings demonstrate that the M protein inhibits IFN production through a SIRT5-dependent mechanism.

Subsequently, we investigated the role of SIRT5 in regulating ROS production during PDCoV infection. CAT, a key peroxisomal enzyme, breaks down hydrogen peroxide (H_2_O_2_) to minimize ROS levels and prevent oxidative damage [57]. PDCoV infection significantly reduced CAT enzyme activity in wild-type cells, but this reduction was absent in SIRT5-KO cells (S6 Fig). To specifically evaluate peroxisomal ROS levels, we used a peroxisome-targeted roGFP2 probe [58]. The correct localization of the roGFP2 probe was verified by immunofluorescence (Fig 5I). This probe allows measurement of ROS levels through oxidation-induced changes in its fluorescence. As shown in Fig 5J, PDCoV infection led to increased ROS levels, which were further elevated by H_2_O_2_ treatment and reduced by treatment with NAC (a ROS scavenger). Importantly, PDCoV-induced peroxisomal ROS levels were lower in SIRT5-KO cells than in wild-type cells. To explore the connection between ROS and PDCoV-induced pexophagy, we treated PDCoV-infected cells with the ROS scavenger NAC. The results showed that NAC treatment inhibited autophagic flux and prevented the PDCoV-induced degradation of peroxisome-associated proteins (Fig 5K). These findings suggest that the PDCoV M protein induces pexophagy, disrupting peroxisomal function and ultimately impacting IFN production and cellular redox balance.

### SIRT5 desuccinylates the PDCoV M protein

**Fig 6:**
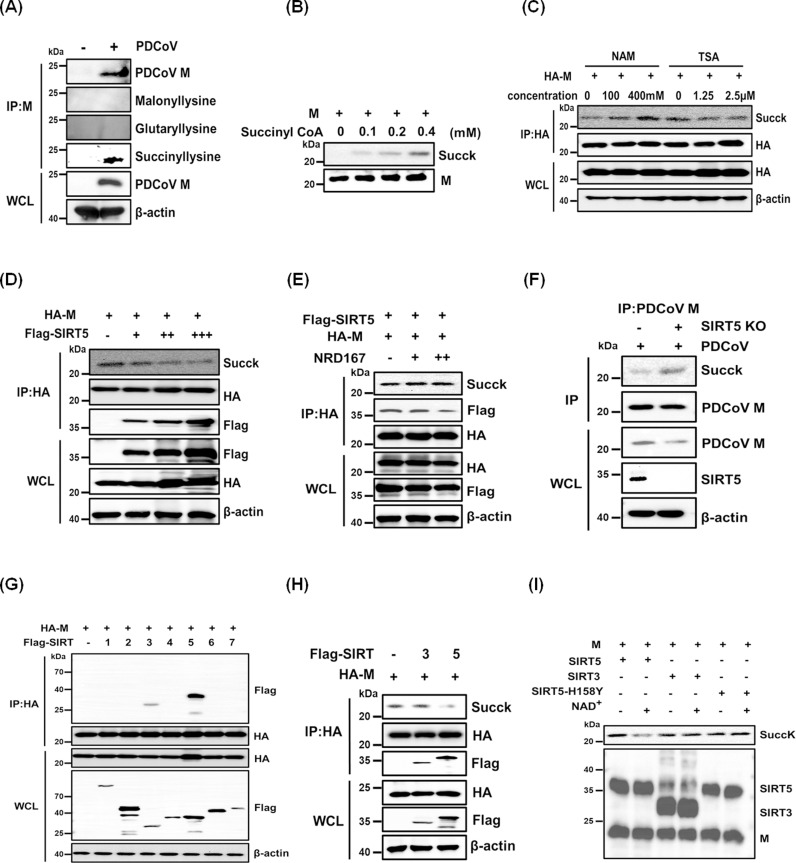
SIRT5 desuccinylates the PDCoV M protein. (**A**) Detection of acylation modifications in the M protein from PDCoV-infected LLC-PK1 cells. (**B**) *In vitro* succinylation assay of the M protein. Purified M proteins were incubated with varying concentrations of succinyl-CoA, and succinylation levels were analyzed by western blotting. (**C**) HEK-293T cells were transfected with pCAGGS-HA-M and treated with different concentrations of NAM or TSA. Cells were collected, and M protein succinylation levels were detected by western blotting. (**D-E**) HEK-293T cells were co-transfected with pCAGGS-HA-M and increasing amounts of pCAGGS-Flag-SIRT5 (**D**) or treated with NRD167 (0, 10, 20 µM) (**E**). Cell lysates were subjected to western blotting. (**F**) SIRT5-KO and wild-type LLC-PK1 cells were infected with PDCoV. Cell lysates were subjected to co-IP using anti-M antibody. WCL and IP complexes were analyzed by western blotting. (**G**) HEK-293T cells were co-transfected with pCAGGS-HA-M and Flag-tagged SIRT1–7 plasmids for 24 h. Cell lysates were subjected to co-IP assays. (**H**) HEK-293T cells were co-transfected with HA-tagged M and Flag-tagged SIRT3 or SIRT5 plasmids for 24 h. Cell lysates were collected and analyzed by co-IP assays. (**I**) Succinylated M proteins were purified from SIRT5-deficient cells and incubated with purified SIRT3, SIRT5, or SIRT5-H158Y proteins in the presence or absence of NAD⁺ at 30°C. After 1 h, the reaction mixtures were analyzed by western blotting.

To determine how SIRT5 regulates M protein-induced pexophagy, we investigated whether SIRT5 influences acyl modifications of the M protein during PDCoV infection. Our analysis revealed that M protein undergoes succinylation but not malonylation or glutarylation in PDCoV-infected cells (Fig 6A). Succinylation is a non-enzymatic process *in vitro*, where succinyl-CoA serves as the active agent for protein modification [59,60]. Thus, we assessed the effect of succinyl-CoA concentration on the succinylation level of M protein *in vitro*, revealing that the succinylation level increased in a succinyl-CoA concentration-dependent manner (Fig 6B). Additionally, M protein succinylation increased in cells treated with NAM (a SIRT inhibitor) but not in cells treated with TSA (a histone deacetylase inhibitor), suggesting that M protein succinylation is regulated by SIRTs, rather than histone deacetylases (Fig 6C). Moreover, SIRT5 reduced the M protein succinylation level in a dose-dependent manner (Fig 6D). When cells co-transfected with HA-M and Flag-SIRT5 were treated with the SIRT5 inhibitor NRD167, the M protein succinylation level was restored (Fig 6E). Furthermore, PDCoV-infected SIRT5-KO cells exhibited higher succinylation levels of M protein compared with wild-type cells (Fig 6F). These results suggested that SIRT5 has a strong desuccinylation effect on, and interacts with, M protein. We also investigated the roles of other SIRTs (1, 2, 3, 4, 6, and 7) in modifying M protein. Among SIRTs tested, only SIRT3 interacted with M protein. However, SIRT3 exhibited weaker desuccinylation activity relative to SIRT5 (Fig 6G and 6H). Notably, only wild-type SIRT5, but not the catalytic mutant SIRT5-H158Y or SIRT3, could desuccinylate M protein *in vitro* (Fig 6I). These results indicate that SIRT5 can desuccinylate M protein both *in vivo* and *in vitro*.

### K207 is the major succinylation site of the PDCoV M protein

**Fig 7:**
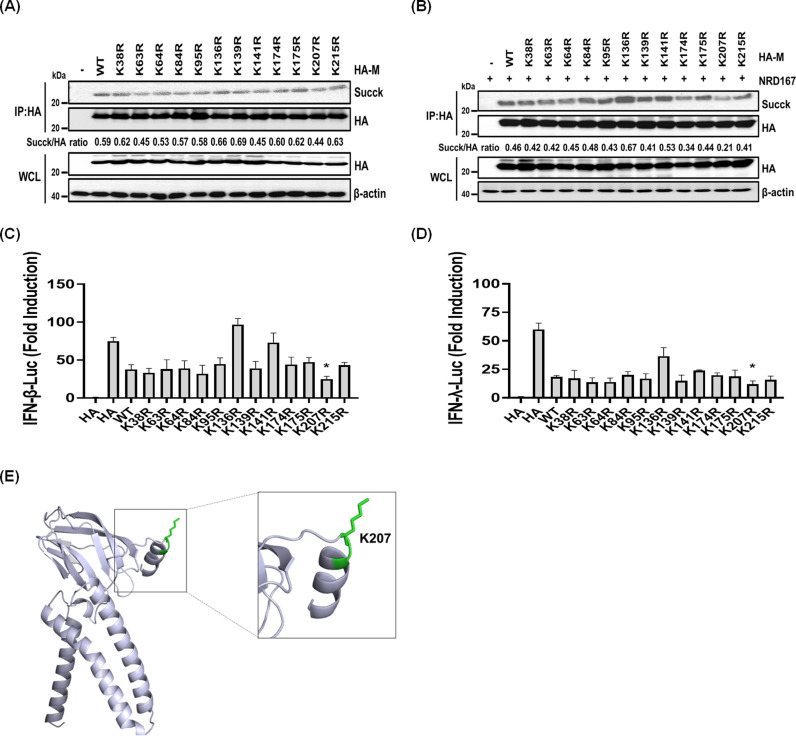
K207 is the major succinylation site of the PDCoV M protein. (**A-B**) HEK-293T cells were transfected with plasmids expressing wild-type M protein or its point mutants for 24 h (A) and further treated with NRD167 (**B**). Cell lysates were analyzed by western blotting to detect M protein succinylation levels. Ratios below images represent the relative M protein succinylation levels, as quantified by ImageJ software. (**C-D**) HEK-293T cells were co-transfected with IFN-β-Luc (C) or IFN-λ-Luc (D) and pRL-TK plasmids for 24 h, along with plasmids expressing wild-type M protein or its point mutants. Cells were then infected with SeV for 12 h, lysed, and analyzed by dual luciferase assays. (**E**) Succinylation sites were mapped onto the crystal structure of the M protein. The succinyl lysine site (K207) is highlighted in the predicted crystal structure of the PDCoV M protein, generated *in silico* using AlphaFold. *p < 0.05. ns, not significant.

To identify succinylation site(s) on M protein, we mutated 12 lysine (K) residues on M protein to arginine (R), mimicking a desuccinylated state. These M protein mutants were overexpressed in cells; succinylated M protein was enriched, and succinylation levels were analyzed by immunoprecipitation (IP). The results showed that succinylation levels substantially decreased when the K207 site was mutated, regardless of NRD167 treatment (Fig 7A and 7B). Next, we assessed the functional impact of the M protein mutants on the production of type I and III IFNs. Compared with the wild-type M protein, the K207R mutant exhibited a notably reduced ability to inhibit IFN production (Fig 7C and 7D). These findings demonstrate that K207 is a critical succinylation site with a key role in M protein function (e.g., regulating IFN production). Mapping of the succinylation site K207 onto the M protein structure revealed that K207 is located on the protein’s surface (Fig 7E). This positioning likely facilitates recognition and desuccinylation by SIRT5, thus influencing M protein functions.

### Desuccinylation of the PDCoV M protein drives pexophagy and enhances viral replication

**Fig 8:**
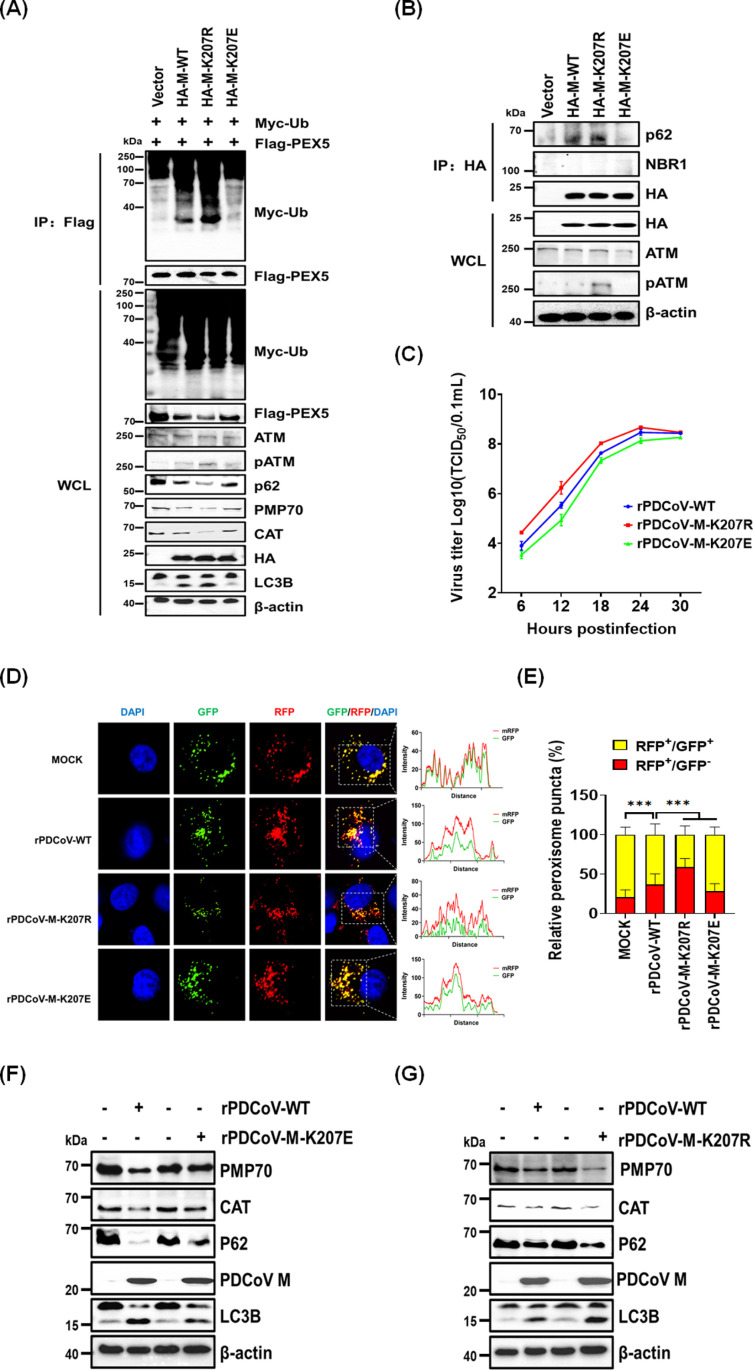
Desuccinylation of the PDCoV M protein drives pexophagy and enhances viral replication. (**A**) HEK-293T cells were co-transfected with pCAGGS-Myc-Ub, pCAGGS-Flag-PEX5, and pCAGGS-HA-M or the indicated M protein mutants for 24 h. Cell lysates were subjected to co-IP using anti-Flag antibody. WCL and IP complexes were analyzed by western blotting with the indicated antibodies. (**B**) HEK-293T cells were transfected with plasmids expressing wild-type M protein or its mutants (K207R, K207E) for 24 h. Cell lysates were subjected to co-IP using anti-HA antibody. WCL and IP complexes were analyzed by western blotting. (C) Multi-step growth curves of rescued rPDCoVs (rPDCoV-WT, rPDCoV-M-K207E, rPDCoV-M-K207R) in LLC-PK1 cells. Cells were infected with rescued rPDCoVs and collected at different post-infection time points (6, 12, 18, 24, 30 hpi) for TCID_50_ assays. (**D-E**) LLC-PK1 cells expressing GFP-RFP-SKL were infected with rescued rPDCoVs, fixed, and analyzed by microscopy to detect colocalization of RFP and GFP signals (**D**). The numbers of RFP^+^/GFP^+^ or RFP^+^/GFP^-^ peroxisomal puncta in rPDCoV-infected cells were quantified using ImageJ software (**E**). (**F-G**) LLC-PK1 cells were infected with rPDCoV-WT, rPDCoV-M-K207E (F), or rPDCoV-M-K207R (G) for 12 h. Cells were harvested, and lysates were analyzed by western blotting with the indicated antibodies.

Given that ROS within peroxisomes can activate ATM, which phosphorylates PEX5 and triggers selective autophagy [61], we explored the role of M protein succinylation in this pathway. HEK-293T cells were co-transfected with plasmids encoding wild-type HA-M (HA-M-WT), the desuccinylated mimic HA-M-K207R, or the succinylated mimic HA-M-K207E (E, mimicking negatively charged succinyl-lysine modification), along with Flag-PEX5 and Myc-Ub. The results revealed that the desuccinylated K207R mutant of M protein increased ATM phosphorylation (pATM) and PEX5 ubiquitination, whereas the succinylated mimic K207E reduced these effects (Fig 8A). Because ubiquitinated peroxisomal proteins can recruit autophagy receptors such as p62 and NBR1 to mediate autophagosome formation [62,63], we analyzed the interactions of M protein with these receptors. We found that M protein specifically interacted with endogenous p62 but not NBR1; this interaction was stronger when M protein was in its desuccinylated state (Fig 8B). Our findings suggest that desuccinylation at K207 facilitates pexophagy by recruiting p62 to ubiquitinated PEX5, promoting autophagic degradation of peroxisomes.

To determine whether desuccinylation at K207 affects viral infection, we generated recombinant PDCoV (rPDCoV) strains with mutations at the K207 site: rPDCoV-M-K207R (mimicking a desuccinylated state) and rPDCoV-M-K207E (mimicking a succinylated state) (S7A and S7B Fig). TCID_50_ assays revealed that rPDCoV-M-K207R exhibited higher viral titers at 6, 12, 18, and 24 h post-infection (hpi) relative to the rescued recombinant wild-type virus (rPDCoV-WT); rPDCoV-M-K207E consistently showed lower viral titers than rPDCoV-M-K207R (Fig 8C). These findings demonstrate that desuccinylation of M protein at K207 enhances viral replication, whereas succinylation at this site inhibits viral replication.

To evaluate the effect of M protein succinylation on pexophagy in the context of PDCoV infection, cells expressing peroxisome-targeted RFP-GFP-SKL were infected with rPDCoV-WT, rPDCoV-M-K207R, or rPDCoV-M-K207E. Compared with rPDCoV-WT, cells infected with rPDCoV-M-K207R exhibited more RFP signals that did not overlap with GFP, whereas cells infected with rPDCoV-M-K207E showed more RFP and GFP double-positive peroxisomal puncta (Fig 8D and 8E). These findings indicate that desuccinylation at the K207 site enhances PDCoV-induced pexophagy. Western blotting analysis confirmed these observations, revealing that rPDCoV-M-K207R induced stronger pexophagy than rPDCoV-WT, while rPDCoV-M-K207E exhibited reduced pexophagy (Fig 8F and 8G). Taken together, these results demonstrate that desuccinylation at the K207 site of the M protein plays a crucial role in enhancing viral replication and mediating pexophagy, whereas succinylation serves as an inhibitory mechanism.

## Discussion

**Fig 9:**
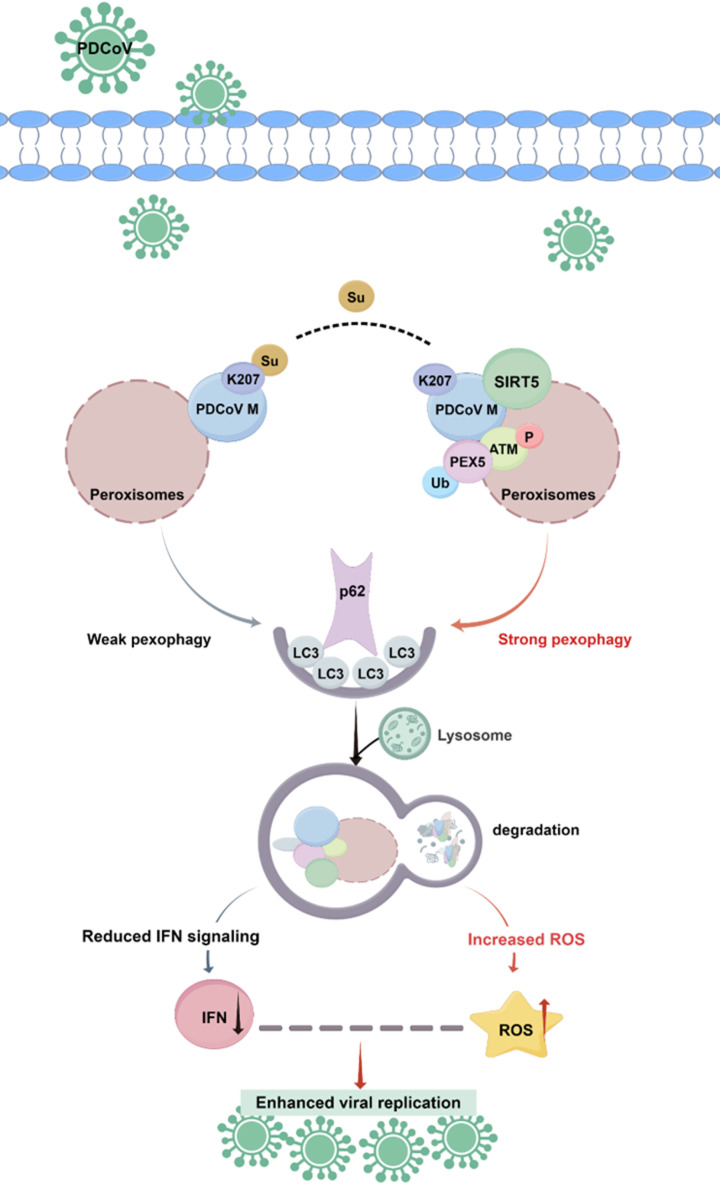
Proposed model for the mechanism by which SIRT5 regulates PDCoV infection. SIRT5 desuccinylates the PDCoV M protein at K207, activating the ATM-PEX5-p62 pathway and inducing pexophagy. This mechanism leads to peroxisome degradation, increased ROS production, and suppression of IFN responses. These combined effects create a favorable environment for enhanced PDCoV replication. This image is drawn by FIGDRAW (www.figdraw.com).

SIRT5, a unique member of the SIRT family, exhibits strong desuccinylation, demalonylation, and deglutarylation activities, along with relatively low deacetylase activity. In this study, we found that SIRT5 interacts with and desuccinylates the PDCoV M protein. This desuccinylation activates the ATM-PEX5-p62 axis, triggering pexophagy. The resulting peroxisome degradation suppresses type I/III interferon production and elevates ROS levels, ultimately promoting viral replication (Fig 9). These findings reveal a novel role for SIRT5 in orchestrating pexophagy during viral infection.

Succinylation, a newly identified post-translational modification (PTM), plays key roles in regulating various physiological processes, such as cellular metabolism and immune responses [64]. Concerning SIRT5-mediated desuccinylation, Gao et al. reported that SIRT5 regulates glycolysis by removing the succinylation of pyruvate kinase M2 [65]. Furthermore, SIRT5-mediated desuccinylation is involved in regulating mitochondrial function. For example, SIRT5 enhances mitochondrial respiratory chain activity through desuccinylation and regulates innate immune responses by removing the succinylation of mitochondrial antiviral signaling protein (MAVS) [54]. In this study, we demonstrated that PDCoV M protein interacts with SIRT5 to suppress type I/III IFN production, providing direct evidence that SIRT5 plays a role in modulating antiviral innate immunity and enhancing viral replication. In addition to M protein, we identified PDCoV nsp14 as an interaction partner of SIRT5, consistent with previous studies that showed interactions between SIRT5 and SARS-CoV-2 nsp14 [40,49]. Furthermore, we also confirmed that PDCoV nsp14 is not a direct substrate of SIRT5-mediated desuccinylation and does not affect pexophagy levels (S8 Fig).

Unlike nsp14, we demonstrated that the PDCoV M protein is a direct substrate of SIRT5, and K207 is the primary succinylation site. However, we also observed that other mutants of the M protein affected succinylation, although to a lesser extent than K207. This finding suggests that, in addition to K207, SIRT5 desuccinylates other lysine residues on the M protein. Investigations of these additional lysine sites and their roles in PDCoV infection will provide a more comprehensive understanding of the involvement of SIRT5 in viral proliferation. Notably, the K207 residue is located on the surface of the PDCoV M protein, a position conducive to PTMs. A comparative analysis with other porcine enteric coronaviruses, including porcine epidemic diarrhea virus, transmissible gastroenteritis virus, and swine acute diarrhea syndrome coronavirus, revealed that K207 is highly conserved and similarly located on the M protein surface (S9 Fig). This conservation of surface exposure suggests that K207 plays an important functional role in the life cycle of porcine enteric coronaviruses. Under physiological conditions, lysine residues typically carry a positive charge. As a PTM, succinylation neutralizes this positive charge, potentially altering the structure and function of the M protein. Specifically, succinylation may regulate M protein activities related to key steps in the viral life cycle, such as replication, assembly, and release. Moreover, a previous study showed that the SARS-CoV-2 M protein degrades MAVS via autophagy to evade the host’s antiviral innate immune responses [66]. Further investigated is warranted concerning whether similar mechanisms are utilized by other coronaviruses.

In our screening of SIRTs that interact with the PDCoV M protein, we identified both SIRT5 and SIRT3 as binding partners. Although SIRT3 inhibits PDCoV replication, whether it directly modifies the M protein remains unclear. SIRT3 primarily catalyzes deacetylation, while SIRT5 is responsible for desuccinylation, demalonylation, and deglutarylation, suggesting that they may regulate distinct PTM sites on M protein and thereby exert different effects on viral replication. Notably, a recent study demonstrated that SIRT3- and SIRT5-mediated modifications of MAVS lysine residues synergistically regulate innate immunity [67], raising the possibility that a similar dual regulatory mechanism may exist for the M protein. Differences in subcellular localization may also influence their interactions with the M protein or other viral replication factors. The interaction of SIRT3 with the M protein could impact mitochondrial functions or signaling pathways distinct from those influenced by SIRT5. Previous research has shown that Newcastle disease virus alters cellular metabolism by shifting from mitochondrial oxidative metabolism to glycolysis through disruption of SIRT3 activity, thus enhancing viral replication [68]. There is a need to explore whether PDCoV utilizes a similar strategy, potentially manipulating SIRT3 and SIRT5 activities to optimize energy metabolism for enhanced replication.

Peroxisomes are essential organelles that participate in lipid metabolism, ROS detoxification, innate immunity, and cellular signaling. It is therefore unsurprising that many viruses disrupt peroxisomal functions to enhance their replication. For example, flavivirus infection impairs peroxisome biogenesis to evade antiviral immune responses [69]. Peroxisomes can be selectively degraded through autophagy, a process known as pexophagy. However, compared with mitophagy, the mechanisms of pexophagy in mammalian cells remain poorly understood [70,71]. Only a few pathways, such as ubiquitination signals, mechanistic target of rapamycin signaling, and elevated ROS levels, have been shown to induce pexophagy [55,72–76]. While numerous studies have explored how viruses utilize autophagy to evade immune surveillance, research into viral regulation of pexophagy remains limited. In this study, we discovered that the PDCoV M protein promotes viral replication by inducing pexophagy, a process dependent on SIRT5-mediated desuccinylation of the M protein. Given that SIRT4 also enhances PDCoV replication, we investigated its role in M-mediated pexophagy. We found that treatment with the SIRT4-specific inhibitor SIRT4‑IN‑1 did not obviously affect PDCoV‑induced pexophagy but significantly suppressed the expressions of TNF‑α, IL‑6, and IL‑8, suggesting that SIRT4 modulates inflammatory response instead of pexophagy (S10 Fig). We also found that the M protein activates the ATM pathway, induces PEX5 ubiquitination, and recruits p62 to facilitate selective peroxisomal autophagy, resulting in peroxisomal dysfunction. Previous study reported that human SIRT5 has a putative peroxisomal targeting signal 2 motif, LQIV, at the N-terminus of human SIRT5, which mediates PEX7-dependent peroxisomal import [43]. Intriguingly, porcine SIRT5 lacks this LQIV motif. Despite this difference, our results showed that porcine SIRT5 interacts with both PEX5 and PEX7 (S11 Fig), implying a distinct peroxisomal targeting mechanism from that of its human ortholog. Moreover, overexpression of key pexophagy-related genes, such as PEX5 and SQSTM1/p62, partially enhanced M protein-induced pexophagy (S12A and S12B Fig), suggesting that both proteins contribute to the regulation of this process. Importantly, inhibition of ATM kinase activity weakened the interaction between M protein and p62 and markedly reduced M-induced pexophagy (S13A and S13B Fig). These findings highlight ATM as a crucial upstream regulator in the M–PEX5–p62 axis, essential for efficient execution of M-mediated pexophagy. Our findings advance the understanding of pexophagy during viral infections and suggest new therapeutic targets for combating coronaviruses, particularly by regulating pexophagy to inhibit viral replication.

In summary, our study reveals a novel role for SIRT5 in regulating PDCoV infection through desuccinylation of the M protein. This modification activates the ATM-PEX5-p62 axis, triggering pexophagy and suppressing type I/III IFN responses, which ultimately enhances viral replication. These findings provide new insights into how SIRT5 modulates virus-host interactions and suggest that targeting SIRT5 could represent a promising therapeutic strategy for controlling PDCoV and related viral infections.

## Materials and methods

### Cell culture and viruses

HEK-293T (ATCC, CRL-11268) and IPI-2I cells (China Center for Type Culture Collection) were cultured in Dulbecco’s Modified Eagle’s Medium supplemented with 10% fetal bovine serum. LLC-PK1 cells were cultured in Modified Eagle’s Medium. The PDCoV strain CHN-HN-2014 (GenBank accession number KT336560), isolated in 2014 from a suckling piglet with severe diarrhea in China [77], was used in this study. SeV was obtained from the Centre of Virus Resource and Information, Wuhan Institute of Virology, Chinese Academy of Sciences, Wuhan, China.

### Antibodies and reagents

Rabbit anti-ATG5 and anti-ATG7monoclonal antibodies (mAbs) were obtained from Hangzhou HuaAn Biotechnology. Rabbit anti-LC3B and anti-PMP70 mAbs were obtained from Abmart. Rabbit anti-Flag, anti-HA-tag and mouse anti-HA, anti-Flag and anti-Myc antibodies were purchased from MBL International Corporation. Rabbit anti-SQSTM1, anti-glutaryllysine and mouse anti-malonyllysine, anti-succinyllysine antibodies were purchased from PTM BioLab. Rabbit anti-PEX5, anti-PEX12, anti-PEX14, anti-CAT, anti-ATM and anti-NBR1 polyclonal antibodies (pAbs) were obtained from Proteintech. Rabbit anti-p-ATM and anti-β-actin pAbs were purchased from ABclonal Technology. Mouse anti-SIRT5 mAb was purchased from Santa Cruz Biotechnology. Mouse anti-PDCoV N protein and anti-PDCoV M protein mAbs were stored in our laboratory [78]. Rapamycin (tlrl-rap) was obtained from InvivoGen. DMSO and CQ were purchased from Sigma-Aldrich. MG132 was purchased from Beyotime Biotechnology. Baf-A1, NAD^+^, KU55933, SIRT4-IN-1 and Torin1 were purchased from MedChemExpress. NRD167, NMN and FK866 were purchased from Selleck.

### Plasmid construction and transfection

Flag-tagged SIRT1–7 and PEXs eukaryotic expression plasmids were constructed by PCR amplification of full-length cDNA from IPI-2I and LLC-PK1 cells, followed by cloning into the pCAGGS vector (MiaoLingBio, P1267). The HA-tagged PDCoV M protein expression plasmid was obtained from our laboratory. Mutant M protein constructs, including substitution mutations M-K38R, -K63R, -K64R, -K84R, -K95R, -K136R, -K139R, -K141R, -K174R, -K175R, -K207R, -K207E and -K215R, were generated by amplifying and cloning the relevant sequences into the pCAGGS-HA-N vector with an HA tag at the N-terminus. All primers used in this study are listed in S1 Table, and the constructed plasmids were validated by DNA sequencing. The RFP-GFP-SKL plasmid was kindly provided by Dr. Min Zhuang (ShanghaiTech University, China). The roGFP2 expression construct was purchased from Addgene (#125582). For transfection experiments, the jetPRIME reagent (Polyplus) was used in accordance with the manufacturer’s protocol.

### Generation of KO cell lines

SIRT5-KO cell lines were generated using the CRISPR/Cas9 system. HEK-293T and LLC-PK1 cells were transfected with the PX459 plasmid containing sgRNAs targeting the SIRT5 gene (S1 Table). After transfection, puromycin selection was performed for 36–48 h. Surviving cells were subcloned by serial dilution in 96-well plates to allow clonal expansion.

### Dual-luciferase reporter assay

Cells grown in 24‐well plates were transfected with various amounts of expression plasmids and the reporter plasmids (IFN-β-Luc or IFN-λ-Luc), as well as with pRL-TK reference plasmid. After 24 h, the luciferase activity was determined by the dual‐luciferase reporter assay system. Representative data from three independently conducted experiments are expressed as the relative firefly luciferase activities with normalization to the Renilla luciferase activities.

### Mitochondria isolation

PDCoV-infected and mock-infected cells were trypsinized and collected by centrifugation at 850 × g for 2 min. Approximately 1 × 10^7^ cells per sample were used for mitochondria isolation with kits from Invent (China; MP-007), in accordance with the manufacturer’s instructions.

### Peroxisome isolation

Peroxisome isolation was performed from LLC-PK1 cells using the Peroxisome Isolation Kit (Sigma, PEROX1). Briefly, 2 × 10⁸ cells were harvested, washed in phosphate-buffered saline, and centrifuged at 250 × g for 5 min. The cell pellet was resuspended in Peroxisome Extraction Buffer (5 mM 3-(N-morpholino) propanesulfonic acid, pH 7.65, 0.25 M sucrose, 1 mM EDTA, 0.1% ethanol, and Protease Inhibitor Cocktail), vortexed, and homogenized with a 7-mL Dounce homogenizer. The homogenate was centrifuged sequentially at 1,000 × g and 2,000 × g for 10 min each. The supernatant was then centrifuged at 25,000 × g for 20 min to isolate the crude peroxisomal fraction, which was further purified by density gradient centrifugation using Optiprep. After centrifugation at 100,000 × g for 1.5 h, the purified peroxisomes were collected for analysis.

### In vitro succinylation assay

Reaction mixtures contained succinylation buffer (20 mM 4-(2-hydroxyethyl)-1-piperazineethanesulfonic acid, pH 8.0, 1 mM dithiothreitol, 1 mM phenylmethyl sulfonyl fluoride, and 0.1 mg/mL bovine serum albumin), purified M proteins, and various concentrations of succinyl-CoA (S1129, Sigma-Aldrich). The mixtures were incubated at 30°C for 15 min. The reactions were stopped by adding loading buffer, then subjected to sodium dodecyl sulfate–polyacrylamide gel electrophoresis. Proteins were analyzed by western blotting.

### In vitro desuccinylation assay

Hypersuccinylated M proteins purified from SIRT5-KO HEK-293T cells were incubated with purified SIRT3, SIRT5, or the enzymatically inactive SIRT5-H158Y in the presence or absence of 1 mM NAD⁺ at 30°C for 1 h. The reaction was performed in desuccinylation buffer [50 mM Tris-HCl (pH 8.0), 100 mM NaCl, 8 mM MgCl₂, 20% glycerol, 1 mM dithiothreitol, 1 mM phenylmethyl sulfonyl fluoride, and 0.1 mg/mL bovine serum albumin]. Proteins were analyzed by western blotting.

### Measurement of ROS in living cells

ROS levels were measured in LLC-PK1 cells transfected with the roGFP2 sensor, as previously described [58]. Briefly, cells were rinsed three times with phosphate-buffered saline before measurement, then placed in phenol red-free Dulbecco’s modified Eagle’s medium. ROS levels were detected using a Cytation5 cell imaging microplate reader (BioTek) and calculated by determining the excitation ratio (405/488). Fluorescence values were background-corrected by subtracting the intensity of samples that did not express the biosensor.

### RNA interference

siRNAs targeting ATG5 (si-ATG5), ATG7 (si-ATG7), or SIRT5 (si-SIRT5), along with negative control siRNA (si-NC), were purchased from Suzhou GenePharma Co., Ltd. siRNA transfection was performed using jetPRIME reagent, in accordance with the manufacturer’s instructions.

### Indirect immunofluorescence assay

The cells were seeded on microscope coverslips in 24-well plates. For examination by immunofluorescence microscopy, the cells were fixed with 4% paraformaldehyde for 10 min at room temperature, permeabilized with methanol for 15 min, blocked in 10% bovine serum albumin for 30 min, and then incubated with primary antibodies. After washes with PBS, the cells were incubated with the secondary antibodies for 1 h at 37°C. Finally, nuclei were stained with DAPI. Fluorescent images were acquired with a confocal laser scanning microscope (Nikon, AXR with NSPARC). Confocal images were analyzed using ImageJ (NIH) software. For colocalization and linescan analysis, images were first split into individual color channels, and regions of interest (ROIs) were selected. Fluorescence intensity profiles were generated using the “Plot Profile” function and saved using the “Save Data” option. For quantification of PEX14-positive puncta, at least 20 cells were randomly selected from the corresponding image panels, and the number of red puncta was manually counted using ImageJ. The total number of puncta per cell was normalized to the MOCK group (set as 100%).

### Generation of recombinant PDCoV

Recombinant PDCoV with single point mutations (K207R or K207E) in the M gene was generated using CRISPR/Cas9, as previously described [78]. Two primers (sgPDCoV-1 and sgPDCoV-2) targeting sequences flanking the mutation site were designed to produce sgRNA-1 and sgRNA-2. The pBAC-CHN-HN-2014 plasmid was linearized by Cas9 cleavage using these sgRNAs. Mutant M gene fragments (K207R or K207E) were generated via overlapping PCR and ligated into the linearized BAC vector through homologous recombination, creating pBAC-CHN-HN-2014-M-K207R and pBAC-CHN-HN-2014-M-K207E. The recombinant plasmids were transfected into LLC-PK1 cells. After 12 h, cells were washed and supplemented with Dulbecco’s Modified Eagle’s Medium containing 7.5 µg/mL trypsin, then incubated at 37°C with 5% CO_2_ for further observation.

### Co-IP assay and western blotting analysis

Cells were transfected with plasmids or infected with virus for the indicated time, then lysed using lysis buffer. For IP, lysates were rotated for 30 min, and a portion of the supernatant was reserved for whole-cell extract assays. The remaining supernatant was immunoprecipitated by incubation with specific antibodies overnight at 4°C, then treated with protein A + G agarose beads (Beyotime Biotechnology, P2055) for 4 h at 4°C. The beads were washed three times with IP lysis buffer. Whole-cell extracts and IP samples were resuspended in sodium dodecyl sulfate–polyacrylamide gel electrophoresis loading buffer. Western blotting analysis was performed using the indicated antibodies.

### Measurement of CAT activity

CAT activity was measured using a Catalase Assay Kit (Beyotime Biotechnology, S0051), in accordance with the manufacturer’s instructions.

### Statistical analysis

Data are presented as mean ± standard deviation (SD). Statistical differences were determined using Student’s *t*-test, one-way analysis of variance, or two-way analysis of variance with GraphPad Prism 8 (GraphPad Inc., USA). Statistical significance thresholds were defined as follows: ns, not significant, *p < 0.05, **p < 0.01, and ***p < 0.001.

## Supporting information

S1 Fig Validation of protein expression. (**A**) The expression of Flag-tagged SIRT family proteins was shown for Fig 1A. (**B**) The expression of SIRT5 was shown for Fig 1D.
(TIF)

S2 Fig Screening of PDCoV-encoded proteins for their potential interaction with SIRT5. (**A**) HEK-293T cells were co-transfected with pCAGGS-Flag-SIRT5 and expression plasmids encoding each individual HA-tagged PDCoV protein. At 24 h post-transfection, the cells were lysed and subjected to Co-IP assay with anti-Flag antibodies. Whole-cell lysate (WCL) and immunoprecipitation (IP) complexes were analyzed by western blotting with antibodies against Flag, HA, or β-actin. (**B**) HEK-293T cells were co-transfected with pCAGGS-Flag-SIRT5 and expression plasmids encoding each of HA-tagged nsp2 and nsp10 for 24 h, followed by Co-IP assay with anti-HA antibodies. WCL and IP complexes were analyzed by western blotting with antibodies against Flag, HA, or β-actin.
(TIF)

S3 Fig Screening peroxisome-associated proteins that interact with PDCoV M protein. HEK-293T cells were co-transfected with pCAGGS-HA-M and expression constructs encoding PEX5, PEX7, PEX11A, PEX11B, PEX11C, PEX12, PEX14, PEX16 or PEX19, respectively for 24 h. The cell lysates were then subjected to co-immunoprecipitation assay with anti-HA antibody and subsequent western blotting.
(TIF)

S4 Fig Overexpression of GFP-SKL can localize to peroxisomes. LLC-PK1 cells were transfected with the GFP-SKL plasmid and then cells were fixed for IFA using anti-PEX14 antibody. Nuclei were counterstained with DAPI. Scale bar, 10 µm.
(TIF)

S5 Fig Construction of SIRT5 KO cell lines. (**A-B**) Western blotting analysis to identify SIRT5 KO HEK-293T cells (A) and LLC-PK1 cells (B).
(TIF)

S6 Fig Detection of catalase activity in PDCoV-infected cells. Catalase activity assay in WT and SIRT5-KO LLC-PK1 cells under mock or PDCoV-infected cells.
(TIF)

S7 Fig Construction of recombinant PDCoV. (**A–B**) Recombinant PDCoV with K207R or K207E mutations in the M gene was generated using CRISPR/Cas9. The sgRNAs targeting sequences flanking the mutation site facilitated Cas9-mediated linearization of the pBAC-CHN-HN-2014 plasmid. Mutant M gene fragments were created via overlapping PCR and inserted into the vector through homologous recombination, yielding recombinant plasmids pBAC-CHN-HN-2014-M-K207R (A) and -K207E (B). Recombinant plasmids were transfected into LLC-PK1 cells, generating recombinant viruses rPDCoV-M-K207R and rPDCoV-M-K207E, respectively.
(TIF)

S8 Fig PDCoV nsp14 is not a direct substrate of SIRT5-mediated desuccinylation and does not affect pexophagy levels. (**A**) HEK-293T cells were transfected with pCAGGS-HA-nsp14 or empty vector, followed by treatment with NRD167 (10 µM). The cells were lysed, and immunoprecipitation was performed using anti-HA antibody. WCL and IP complexes were analyzed by western blotting. (**B**) HEK-293T cells were co-transfected with pCAGGS-HA-nsp14 and pCAGGS-Flag-SIRT5, along with empty vector controls. The cells lysates were subjected to co-immunoprecipitation assay with anti-HA antibody and subsequent western blotting. (**C**) HEK-293T cells were transfected with pCAGGS-HA-nsp14 at increasing doses, then collected for western blotting analysis.
(TIF)

S9 Fig Conservation analysis of the K207 locus of porcine enteric coronavirus M protein. (**A**) Alignment of the amino acid sequences of the M proteins of PDCoV, SADS-CoV, PEDV and TGEV. The lysine 207 site of the porcine coronavirus M protein is indicated by a pentagram. (**B-C**) The three-dimensional structures of PDCoV M (yellow), SADS-CoV M (blue), PEDV M (green), and TGEV M (purple) obtained from Alpha Fold were analyzed with PyMOL software.
(TIF)

S10 Fig SIRT4 modulates inflammatory response instead of pexophagy. (**A)** LLC-PK1 cells were treated with SIRT4-IN-1 (100 µM) or left untreated, then infected with PDCoV for 12 h. Cells were harvested, and lysates were analyzed by western blotting with the indicated antibodies. (**B**) LLC-PK1 cells were treated with SIRT4-IN-1 (100 µM) or left untreated, then infected with PDCoV for 12 h. The cells were collected and subjected to RT-qPCR.
(TIF)

S11 Fig Porcine SIRT5 interacts with both PEX5 and PEX7. (**A**) Sequence alignment of N-terminal regions of porcine and human SIRT5, highlighting the divergent residues. (**B**) HEK-293T cells were co-transfected with pCAGGS-HA-SIRT5 and expression constructs encoding Flag-PEX5, PEX7, PEX12, or PEX14 for 24 h. Cell lysates were subjected to co-immunoprecipitation with anti-HA antibody, followed by western blotting analysis.
(TIF)

S12 Fig PEX5 or p62 contributes to the regulation of M protein-induced pexophagy. HEK-293T cells were co-transfected with pCAGGS-HA-M and either pCAGGS-Flag-PEX5 (**A**) or pCAGGS-Flag-p62 (**B**), along with empty vector controls. After 24 h, cell lysates were subjected to western blotting to detect the expression of HA-tagged M protein, Flag-tagged PEX5 or p62, and peroxisomal markers (PMP70, CAT), as well as LC3B as an autophagy marker.
(TIF)

S13 Fig ATM kinase activity is essential for M-mediated pexophagy. (**A**) HEK-293T cells were transfected with pCAGGS-HA-M or empty vector, followed by treatment with KU-55933 (10 µM). The cells lysates were subjected to co-immunoprecipitation assay with anti-HA antibody and subsequent western blotting. (**B**) HEK-293T cells were treated as in (A). Total cell lysates were analyzed by western blotting.
(TIF)

S1 Table The primers used for plasmid constructions and sequences of siRNA used in this study. (XLSX)

S1 Data Excel spreadsheet containing the underlying numerical data for Figs 1A-H, 4J, 5C, 5F, 5G, 5H, 5J, 7C, 8C, 8E, S6, S10B in separate sheets. (XLSX)

## References

[1] Li W, Hulswit RJG, Kenney SP, Widjaja I, Jung K, Alhamo MA, et al. Broad receptor engagement of an emerging global coronavirus may potentiate its diverse cross-species transmissibility. Proc Natl Acad Sci U S A. 2018;115(22):E5135–43. doi: 10.1073/pnas.1802879115 29760102 PMC5984533

[2] Jung K, Hu H, Saif LJ. Porcine deltacoronavirus infection: Etiology, cell culture for virus isolation and propagation, molecular epidemiology and pathogenesis. Virus Res. 2016;226:50–9. doi: 10.1016/j.virusres.2016.04.009 27086031 PMC7114557

[3] Woo PCY, Lau SKP, Lam CSF, Lau CCY, Tsang AKL, Lau JHN, et al. Discovery of seven novel Mammalian and avian coronaviruses in the genus deltacoronavirus supports bat coronaviruses as the gene source of alphacoronavirus and betacoronavirus and avian coronaviruses as the gene source of gammacoronavirus and deltacoronavirus. J Virol. 2012;86(7):3995–4008. doi: 10.1128/JVI.06540-11 22278237 PMC3302495

[4] Lorsirigool A, Saeng-Chuto K, Madapong A, Temeeyasen G, Tripipat T, Kaewprommal P, et al. The genetic diversity and complete genome analysis of two novel porcine deltacoronavirus isolates in Thailand in 2015. Virus Genes. 2017;53(2):240–8. doi: 10.1007/s11262-016-1413-z 28005234

[5] Dong N, Fang L, Zeng S, Sun Q, Chen H, Xiao S. Porcine Deltacoronavirus in Mainland China. Emerg Infect Dis. 2015;21(12):2254–5. doi: 10.3201/eid2112.150283 26584185 PMC4672429

[6] Jang G, Lee K-K, Kim S-H, Lee C. Prevalence, complete genome sequencing and phylogenetic analysis of porcine deltacoronavirus in South Korea, 2014-2016. Transbound Emerg Dis. 2017;64(5):1364–70. doi: 10.1111/tbed.12690 28758347 PMC7169712

[7] Saeng-Chuto K, Lorsirigool A, Temeeyasen G, Vui DT, Stott CJ, Madapong A, et al. Different Lineage of Porcine Deltacoronavirus in Thailand, Vietnam and Lao PDR in 2015. Transbound Emerg Dis. 2017;64(1):3–10. doi: 10.1111/tbed.12585 27718337 PMC7169859

[8] Xu Z, Zhong H, Zhou Q, Du Y, Chen L, Zhang Y, et al. A Highly Pathogenic Strain of Porcine Deltacoronavirus Caused Watery Diarrhea in Newborn Piglets. Virol Sin. 2018;33(2):131–41. doi: 10.1007/s12250-018-0003-8 29569144 PMC6178105

[9] Suzuki T, Shibahara T, Imai N, Yamamoto T, Ohashi S. Genetic characterization and pathogenicity of Japanese porcine deltacoronavirus. Infect Genet Evol. 2018;61:176–82. doi: 10.1016/j.meegid.2018.03.030 29621617 PMC7172274

[10] Turlewicz-Podbielska H, Pomorska-Mól M. Porcine Coronaviruses: Overview of the State of the Art. Virol Sin. 2021;36(5):833–51. doi: 10.1007/s12250-021-00364-0 33723809 PMC7959302

[11] Boley PA, Alhamo MA, Lossie G, Yadav KK, Vasquez-Lee M, Saif LJ, et al. Porcine Deltacoronavirus Infection and Transmission in Poultry, United States1. Emerg Infect Dis. 2020;26(2):255–65. doi: 10.3201/eid2602.190346 31961296 PMC6986833

[12] Jung K, Hu H, Saif LJ. Calves are susceptible to infection with the newly emerged porcine deltacoronavirus, but not with the swine enteric alphacoronavirus, porcine epidemic diarrhea virus. Arch Virol. 2017;162(8):2357–62. doi: 10.1007/s00705-017-3351-z 28374120 PMC7086908

[13] Liang Q, Zhang H, Li B, Ding Q, Wang Y, Gao W, et al. Susceptibility of Chickens to Porcine Deltacoronavirus Infection. Viruses. 2019;11(6):573. doi: 10.3390/v11060573 31234434 PMC6631122

[14] Liu Y, Wang B, Liang Q-Z, Shi F-S, Ji C-M, Yang X-L, et al. Roles of Two Major Domains of the Porcine Deltacoronavirus S1 Subunit in Receptor Binding and Neutralization. J Virol. 2021;95(24):e0111821. doi: 10.1128/JVI.01118-21 34549985 PMC8610578

[15] Lednicky JA, Tagliamonte MS, White SK, Elbadry MA, Alam MM, Stephenson CJ, et al. Independent infections of porcine deltacoronavirus among Haitian children. Nature. 2021;600(7887):133–7. doi: 10.1038/s41586-021-04111-z 34789872 PMC8636265

[16] Zhai S-L, Sun M-F, Zhang J-F, Zheng C, Liao M. Spillover infection of common animal coronaviruses to humans. Lancet Microbe. 2022;3(11):e808. doi: 10.1016/S2666-5247(22)00198-7 35878623

[17] Sawicki SG, Sawicki DL, Younker D, Meyer Y, Thiel V, Stokes H, et al. Functional and genetic analysis of coronavirus replicase-transcriptase proteins. PLoS Pathog. 2005;1(4):e39. doi: 10.1371/journal.ppat.0010039 16341254 PMC1298938

[18] Fang P, Fang L, Liu X, Hong Y, Wang Y, Dong N, et al. Identification and subcellular localization of porcine deltacoronavirus accessory protein NS6. Virology. 2016;499:170–7. doi: 10.1016/j.virol.2016.09.015 27661736 PMC7111631

[19] Shen L, Bard JD, Triche TJ, Judkins AR, Biegel JA, Gai X. Emerging variants of concern in SARS-CoV-2 membrane protein: a highly conserved target with potential pathological and therapeutic implications. Emerg Microbes Infect. 2021;10(1):885–93. doi: 10.1080/22221751.2021.1922097 33896413 PMC8118436

[20] Sato T, Takeyama N, Katsumata A, Tuchiya K, Kodama T, Kusanagi K. Mutations in the spike gene of porcine epidemic diarrhea virus associated with growth adaptation in vitro and attenuation of virulence in vivo. Virus Genes. 2011;43(1):72–8. doi: 10.1007/s11262-011-0617-5 21559974 PMC7088782

[21] Zhang Z, Chen J, Shi H, Chen X, Shi D, Feng L, et al. Identification of a conserved linear B-cell epitope in the M protein of porcine epidemic diarrhea virus. Virol J. 2012;9:225. doi: 10.1186/1743-422X-9-225 23025700 PMC3519612

[22] Arndt AL, Larson BJ, Hogue BG. A conserved domain in the coronavirus membrane protein tail is important for virus assembly. J Virol. 2010;84(21):11418–28. doi: 10.1128/JVI.01131-10 20719948 PMC2953170

[23] Fan J-H, Zuo Y-Z, Li J-H, Pei L-H. Heterogeneity in membrane protein genes of porcine epidemic diarrhea viruses isolated in China. Virus Genes. 2012;45(1):113–7. doi: 10.1007/s11262-012-0755-4 22585338 PMC7088567

[24] Imai S-I, Guarente L. It takes two to tango: NAD+ and sirtuins in aging/longevity control. NPJ Aging Mech Dis. 2016;2:16017. doi: 10.1038/npjamd.2016.17 28721271 PMC5514996

[25] Houtkooper RH, Pirinen E, Auwerx J. Sirtuins as regulators of metabolism and healthspan. Nat Rev Mol Cell Biol. 2012;13(4):225–38. doi: 10.1038/nrm3293 22395773 PMC4872805

[26] Vaquero A, Scher MB, Lee DH, Sutton A, Cheng H-L, Alt FW, et al. SirT2 is a histone deacetylase with preference for histone H4 Lys 16 during mitosis. Genes Dev. 2006;20(10):1256–61. doi: 10.1101/gad.1412706 16648462 PMC1472900

[27] Ford E, Voit R, Liszt G, Magin C, Grummt I, Guarente L. Mammalian Sir2 homolog SIRT7 is an activator of RNA polymerase I transcription. Genes Dev. 2006;20(9):1075–80. doi: 10.1101/gad.1399706 16618798 PMC1472467

[28] Michishita E, Park JY, Burneskis JM, Barrett JC, Horikawa I. Evolutionarily conserved and nonconserved cellular localizations and functions of human SIRT proteins. Mol Biol Cell. 2005;16(10):4623–35. doi: 10.1091/mbc.e05-01-0033 16079181 PMC1237069

[29] Rack JGM, VanLinden MR, Lutter T, Aasland R, Ziegler M. Constitutive nuclear localization of an alternatively spliced sirtuin-2 isoform. J Mol Biol. 2014;426(8):1677–91. doi: 10.1016/j.jmb.2013.10.027 24177535

[30] Onyango P, Celic I, McCaffery JM, Boeke JD, Feinberg AP. SIRT3, a human SIR2 homologue, is an NAD-dependent deacetylase localized to mitochondria. Proc Natl Acad Sci U S A. 2002;99(21):13653–8. doi: 10.1073/pnas.222538099 12374852 PMC129731

[31] Tomaselli D, Steegborn C, Mai A, Rotili D. Sirt4: A Multifaceted Enzyme at the Crossroads of Mitochondrial Metabolism and Cancer. Front Oncol. 2020;10:474. doi: 10.3389/fonc.2020.00474 32373514 PMC7177044

[32] Taurone S, De Ponte C, Rotili D, De Santis E, Mai A, Fiorentino F, et al. Biochemical Functions and Clinical Characterizations of the Sirtuins in Diabetes-Induced Retinal Pathologies. Int J Mol Sci. 2022;23(7):4048. doi: 10.3390/ijms23074048 35409409 PMC8999941

[33] Roessler C, Tüting C, Meleshin M, Steegborn C, Schutkowski M. A Novel Continuous Assay for the Deacylase Sirtuin 5 and Other Deacetylases. J Med Chem. 2015;58(18):7217–23. doi: 10.1021/acs.jmedchem.5b00293 26308971

[34] Du J, Zhou Y, Su X, Yu JJ, Khan S, Jiang H, et al. Sirt5 is a NAD-dependent protein lysine demalonylase and desuccinylase. Science. 2011;334(6057):806–9. doi: 10.1126/science.1207861 22076378 PMC3217313

[35] Shi L, Yan H, An S, Shen M, Jia W, Zhang R, et al. SIRT5-mediated deacetylation of LDHB promotes autophagy and tumorigenesis in colorectal cancer. Mol Oncol. 2019;13(2):358–75. doi: 10.1002/1878-0261.12408 30443978 PMC6360364

[36] Gu W, Qian Q, Xu Y, Xu X, Zhang L, He S, et al. SIRT5 regulates autophagy and apoptosis in gastric cancer cells. J Int Med Res. 2021;49(2):300060520986355. doi: 10.1177/0300060520986355 33530803 PMC7871096

[37] Garva R, Thepmalee C, Yasamut U, Sudsaward S, Guazzelli A, Rajendran R, et al. Sirtuin Family Members Selectively Regulate Autophagy in Osteosarcoma and Mesothelioma Cells in Response to Cellular Stress. Front Oncol. 2019;9:949. doi: 10.3389/fonc.2019.00949 31608237 PMC6771295

[38] Polletta L, Vernucci E, Carnevale I, Arcangeli T, Rotili D, Palmerio S, et al. SIRT5 regulation of ammonia-induced autophagy and mitophagy. Autophagy. 2015;11(2):253–70. doi: 10.1080/15548627.2015.1009778 25700560 PMC4502726

[39] Li Z, Zheng Z, Dai X. SIRT5 induces autophagy and alleviates myocardial infarction via desuccinylation of TOM1. BMC Cardiovasc Disord. 2024;24(1):464. doi: 10.1186/s12872-024-04120-6 39210272 PMC11363360

[40] Walter M, Chen IP, Vallejo-Gracia A, Kim I-J, Bielska O, Lam VL, et al. SIRT5 is a proviral factor that interacts with SARS-CoV-2 Nsp14 protein. PLoS Pathog. 2022;18(9):e1010811. doi: 10.1371/journal.ppat.1010811 36095012 PMC9499238

[41] Gordon DE, Jang GM, Bouhaddou M, Xu J, Obernier K, White KM, et al. A SARS-CoV-2 protein interaction map reveals targets for drug repurposing. Nature. 2020;583(7816):459–68. doi: 10.1038/s41586-020-2286-9 32353859 PMC7431030

[42] Nishida Y, Rardin MJ, Carrico C, He W, Sahu AK, Gut P, et al. SIRT5 Regulates both Cytosolic and Mitochondrial Protein Malonylation with Glycolysis as a Major Target. Mol Cell. 2015;59(2):321–32. doi: 10.1016/j.molcel.2015.05.022 26073543 PMC4571487

[43] Chen X-F, Tian M-X, Sun R-Q, Zhang M-L, Zhou L-S, Jin L, et al. SIRT5 inhibits peroxisomal ACOX1 to prevent oxidative damage and is downregulated in liver cancer. EMBO Rep. 2018;19(5):e45124. doi: 10.15252/embr.201745124 29491006 PMC5934778

[44] Odendall C, Dixit E, Stavru F, Bierne H, Franz KM, Durbin AF, et al. Diverse intracellular pathogens activate type III interferon expression from peroxisomes. Nat Immunol. 2014;15(8):717–26. doi: 10.1038/ni.2915 24952503 PMC4106986

[45] Dixit E, Boulant S, Zhang Y, Lee ASY, Odendall C, Shum B, et al. Peroxisomes are signaling platforms for antiviral innate immunity. Cell. 2010;141(4):668–81. doi: 10.1016/j.cell.2010.04.018 20451243 PMC3670185

[46] Steinberg SJ, Dodt G, Raymond GV, Braverman NE, Moser AB, Moser HW. Peroxisome biogenesis disorders. Biochim Biophys Acta. 2006;1763(12):1733–48. doi: 10.1016/j.bbamcr.2006.09.010 17055079

[47] Liu X, Ma C, Subramani S. Recent advances in peroxisomal matrix protein import. Curr Opin Cell Biol. 2012;24(4):484–9. doi: 10.1016/j.ceb.2012.05.003 22683191 PMC3425728

[48] Manjithaya R, Nazarko TY, Farré J-C, Subramani S. Molecular mechanism and physiological role of pexophagy. FEBS Lett. 2010;584(7):1367–73. doi: 10.1016/j.febslet.2010.01.019 20083110 PMC2843806

[49] Liu Q, Wang H, Zhang H, Sui L, Li L, Xu W, et al. The global succinylation of SARS-CoV-2-infected host cells reveals drug targets. Proc Natl Acad Sci U S A. 2022;119(30):e2123065119. doi: 10.1073/pnas.2123065119 35858407 PMC9335334

[50] Hu J, Chen J, Hou Q, Xu X, Ren J, Ma L, et al. Core-predominant gut fungus Kazachstania slooffiae promotes intestinal epithelial glycolysis via lysine desuccinylation in pigs. Microbiome. 2023;11(1):31. doi: 10.1186/s40168-023-01468-3 36814349 PMC9948344

[51] Tan M, Peng C, Anderson KA, Chhoy P, Xie Z, Dai L, et al. Lysine glutarylation is a protein posttranslational modification regulated by SIRT5. Cell Metab. 2014;19(4):605–17. doi: 10.1016/j.cmet.2014.03.014 24703693 PMC4108075

[52] Verdin E. NAD⁺ in aging, metabolism, and neurodegeneration. Science. 2015;350(6265):1208–13. doi: 10.1126/science.aac4854 26785480

[53] Covarrubias AJ, Kale A, Perrone R, Lopez-Dominguez JA, Pisco AO, Kasler HG, et al. Senescent cells promote tissue NAD+ decline during ageing via the activation of CD38+ macrophages. Nat Metab. 2020;2(11):1265–83. doi: 10.1038/s42255-020-00305-3 33199924 PMC7908681

[54] Liu X, Zhu C, Zha H, Tang J, Rong F, Chen X, et al. SIRT5 impairs aggregation and activation of the signaling adaptor MAVS through catalyzing lysine desuccinylation. EMBO J. 2020;39(11):e103285. doi: 10.15252/embj.2019103285 32301534 PMC7265249

[55] Zheng J, Chen X, Liu Q, Zhong G, Zhuang M. Ubiquitin ligase MARCH5 localizes to peroxisomes to regulate pexophagy. J Cell Biol. 2022;221(1):e202103156. doi: 10.1083/jcb.202103156 34747980 PMC8579195

[56] Smith JJ, Aitchison JD. Peroxisomes take shape. Nat Rev Mol Cell Biol. 2013;14(12):803–17. doi: 10.1038/nrm3700 24263361 PMC4060825

[57] Walker CL, Pomatto LCD, Tripathi DN, Davies KJA. Redox Regulation of Homeostasis and Proteostasis in Peroxisomes. Physiol Rev. 2018;98(1):89–115. doi: 10.1152/physrev.00033.2016 29167332 PMC6335096

[58] Ivashchenko O, Van Veldhoven PP, Brees C, Ho Y-S, Terlecky SR, Fransen M. Intraperoxisomal redox balance in mammalian cells: oxidative stress and interorganellar cross-talk. Mol Biol Cell. 2011;22(9):1440–51. doi: 10.1091/mbc.E10-11-0919 21372177 PMC3084667

[59] Wagner GR, Payne RM. Widespread and enzyme-independent Nε-acetylation and Nε-succinylation of proteins in the chemical conditions of the mitochondrial matrix. J Biol Chem. 2013;288(40):29036–45. doi: 10.1074/jbc.M113.486753 23946487 PMC3790002

[60] Wagner GR, Bhatt DP, O’Connell TM, Thompson JW, Dubois LG, Backos DS, et al. A Class of Reactive Acyl-CoA Species Reveals the Non-enzymatic Origins of Protein Acylation. Cell Metab. 2017;25(4):823-837.e8. doi: 10.1016/j.cmet.2017.03.006 28380375 PMC5399522

[61] Zhang J, Tripathi DN, Jing J, Alexander A, Kim J, Powell RT, et al. ATM functions at the peroxisome to induce pexophagy in response to ROS. Nat Cell Biol. 2015;17(10):1259–69. doi: 10.1038/ncb3230 26344566 PMC4589490

[62] Kim PK, Hailey DW, Mullen RT, Lippincott-Schwartz J. Ubiquitin signals autophagic degradation of cytosolic proteins and peroxisomes. Proc Natl Acad Sci U S A. 2008;105(52):20567–74. doi: 10.1073/pnas.0810611105 19074260 PMC2602605

[63] Deosaran E, Larsen KB, Hua R, Sargent G, Wang Y, Kim S, et al. NBR1 acts as an autophagy receptor for peroxisomes. J Cell Sci. 2013;126(Pt 4):939–52. doi: 10.1242/jcs.114819 23239026

[64] Zhang Z, Tan M, Xie Z, Dai L, Chen Y, Zhao Y. Identification of lysine succinylation as a new post-translational modification. Nat Chem Biol. 2011;7(1):58–63. doi: 10.1038/nchembio.495 21151122 PMC3065206

[65] Gao X, Wang H, Yang JJ, Liu X, Liu Z-R. Pyruvate kinase M2 regulates gene transcription by acting as a protein kinase. Mol Cell. 2012;45(5):598–609. doi: 10.1016/j.molcel.2012.01.001 22306293 PMC3299833

[66] Hui X, Zhang L, Cao L, Huang K, Zhao Y, Zhang Y, et al. SARS-CoV-2 promote autophagy to suppress type I interferon response. Signal Transduct Target Ther. 2021;6(1):180. doi: 10.1038/s41392-021-00574-8 33966045 PMC8105701

[67] Liu X, Zhu C, Jia S, Deng H, Tang J, Sun X, et al. Dual modifying of MAVS at lysine 7 by SIRT3-catalyzed deacetylation and SIRT5-catalyzed desuccinylation orchestrates antiviral innate immunity. Proc Natl Acad Sci U S A. 2024;121(17):e2314201121. doi: 10.1073/pnas.2314201121 38635631 PMC11047105

[68] Gong Y, Tang N, Liu P, Sun Y, Lu S, Liu W, et al. Newcastle disease virus degrades SIRT3 via PINK1-PRKN-dependent mitophagy to reprogram energy metabolism in infected cells. Autophagy. 2022;18(7):1503–21. doi: 10.1080/15548627.2021.1990515 34720029 PMC9298456

[69] You J, Hou S, Malik-Soni N, Xu Z, Kumar A, Rachubinski RA, et al. Flavivirus Infection Impairs Peroxisome Biogenesis and Early Antiviral Signaling. J Virol. 2015;89(24):12349–61. doi: 10.1128/JVI.01365-15 26423946 PMC4665241

[70] Gatica D, Lahiri V, Klionsky DJ. Cargo recognition and degradation by selective autophagy. Nat Cell Biol. 2018;20(3):233–42. doi: 10.1038/s41556-018-0037-z 29476151 PMC6028034

[71] Grumati P, Dikic I. Ubiquitin signaling and autophagy. J Biol Chem. 2018;293(15):5404–13. doi: 10.1074/jbc.TM117.000117 29187595 PMC5900779

[72] Wilhelm LP, Zapata-Muñoz J, Villarejo-Zori B, Pellegrin S, Freire CM, Toye AM, et al. BNIP3L/NIX regulates both mitophagy and pexophagy. EMBO J. 2022;41(24):e111115. doi: 10.15252/embj.2022111115 36215693 PMC9753467

[73] Demers ND, Riccio V, Jo DS, Bhandari S, Law KB, Liao W, et al. PEX13 prevents pexophagy by regulating ubiquitinated PEX5 and peroxisomal ROS. Autophagy. 2023;19(6):1781–802. doi: 10.1080/15548627.2022.2160566 36541703 PMC10262789

[74] Sargent G, van Zutphen T, Shatseva T, Zhang L, Di Giovanni V, Bandsma R, et al. PEX2 is the E3 ubiquitin ligase required for pexophagy during starvation. J Cell Biol. 2016;214(6):677–90. doi: 10.1083/jcb.201511034 27597759 PMC5021090

[75] Germain K, Kim PK. Pexophagy: A Model for Selective Autophagy. Int J Mol Sci. 2020;21(2):578. doi: 10.3390/ijms21020578 31963200 PMC7013971

[76] Tripathi DN, Zhang J, Jing J, Dere R, Walker CL. A new role for ATM in selective autophagy of peroxisomes (pexophagy). Autophagy. 2016;12(4):711–2. doi: 10.1080/15548627.2015.1123375 27050462 PMC4836024

[77] Dong N, Fang L, Yang H, Liu H, Du T, Fang P, et al. Isolation, genomic characterization, and pathogenicity of a Chinese porcine deltacoronavirus strain CHN-HN-2014. Vet Microbiol. 2016;196:98–106. doi: 10.1016/j.vetmic.2016.10.022 27939164 PMC7117368

[78] Li Z, Duan P, Qiu R, Fang L, Fang P, Xiao S. HDAC6 Degrades nsp8 of Porcine Deltacoronavirus through Deacetylation and Ubiquitination to Inhibit Viral Replication. J Virol. 2023;97(5):e0037523. doi: 10.1128/jvi.00375-23 37133375 PMC10231189

