# Supplementary figures and images for: SIRT5-mediated desuccinylation of the porcine deltacoronavirus M protein drives pexophagy to enhance viral proliferation
Source: PLoS Pathog. 2025 May 9;21(5):e1013163. doi: 10.1371/journal.ppat.1013163 (PMC12143549; doi:10.1371/journal.ppat.1013163)

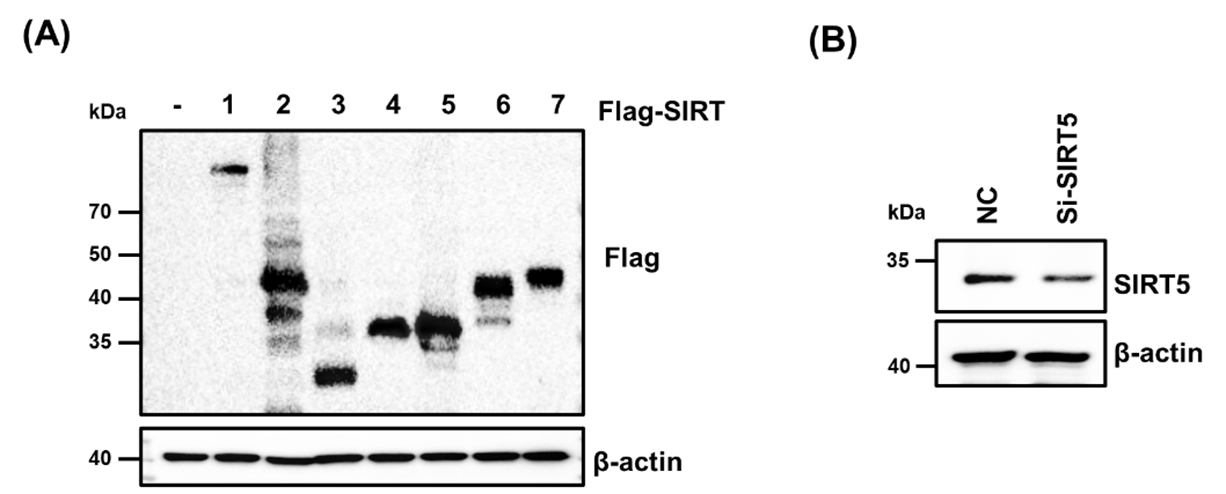

Supplement: S1 Fig — (A) The expression of Flag-tagged SIRT family proteins was shown for Fig 1A. (B) The expression of SIRT5 was shown for Fig 1D. (TIF) [file ppat.1013163.s001.tif]

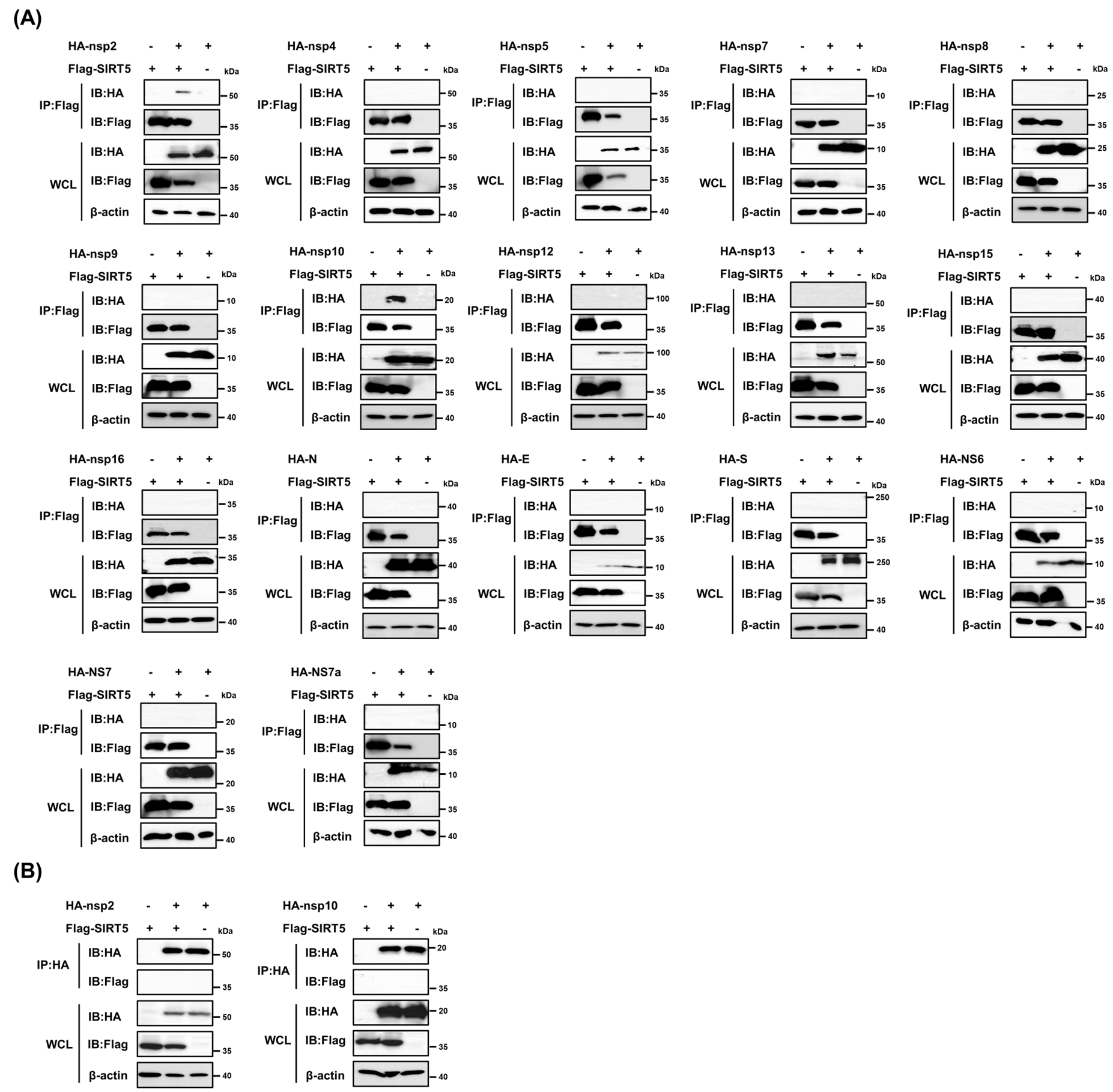

Supplement: S2 Fig — (A) HEK-293T cells were co-transfected with pCAGGS-Flag-SIRT5 and expression plasmids encoding each individual HA-tagged PDCoV protein. At 24 h post-transfection, the cells were lysed and subjected to Co-IP assay with anti-Flag antibodies. Whole-cell lysate (WCL) and immunoprecipitation (IP) complexes were analyzed by western blotting with antibodies against Flag, HA, or β-actin. (B) HEK-293T cells were co-transfected with pCAGGS-Flag-SIRT5 and expression plasmids encoding each of HA-tagged nsp2 and nsp10 for 24 h, followed by Co-IP assay with anti-HA antibodies. WCL and IP complexes were analyzed by western blotting with antibodies against Flag, HA, or β-actin. (TIF) [file ppat.1013163.s002.tif]

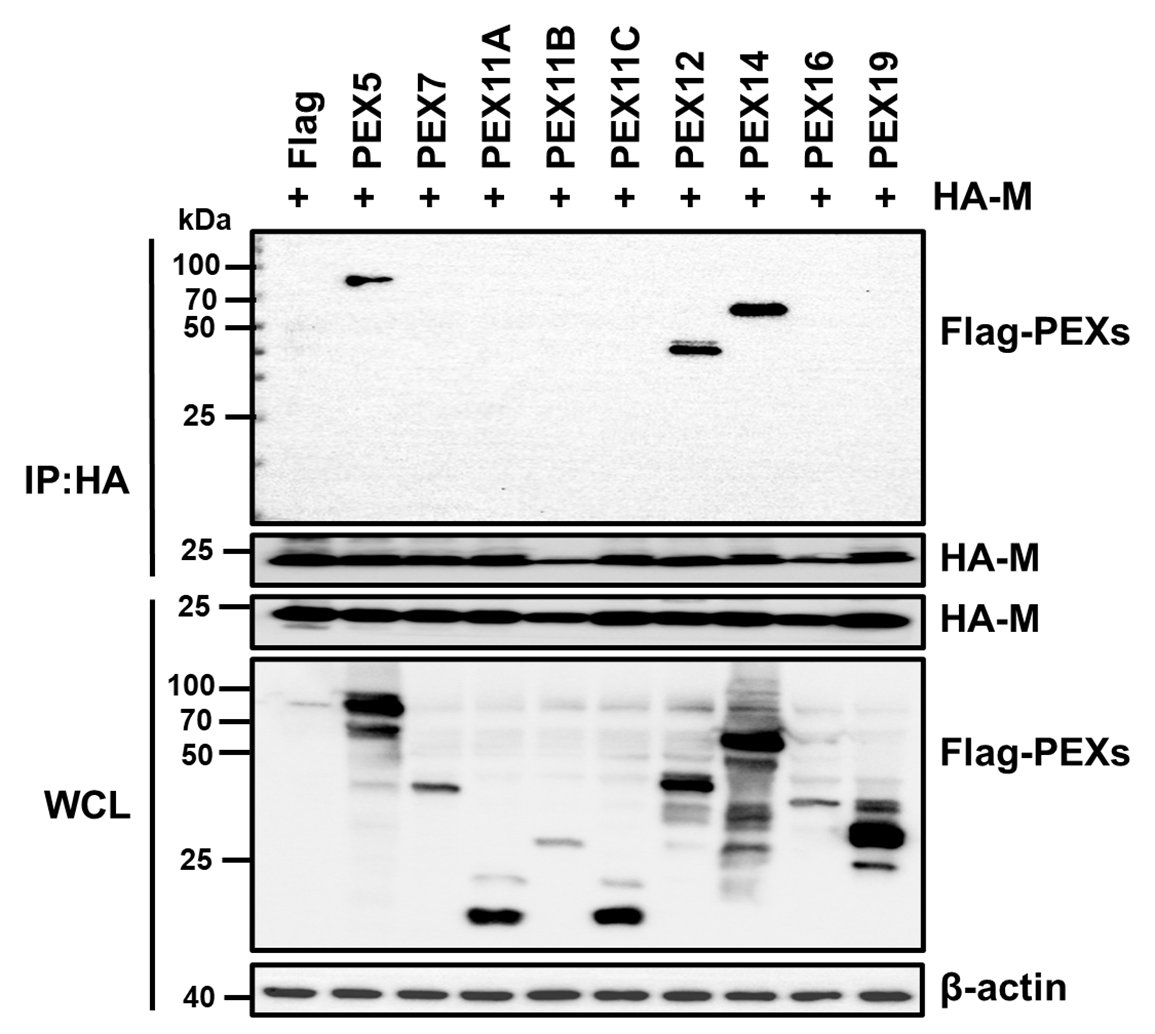

Supplement: S3 Fig — HEK-293T cells were co-transfected with pCAGGS-HA-M and expression constructs encoding PEX5, PEX7, PEX11A, PEX11B, PEX11C, PEX12, PEX14, PEX16 or PEX19, respectively for 24 h. The cell lysates were then subjected to co-immunoprecipitation assay with anti-HA antibody and subsequent western blotting. (TIF) [file ppat.1013163.s003.tif]

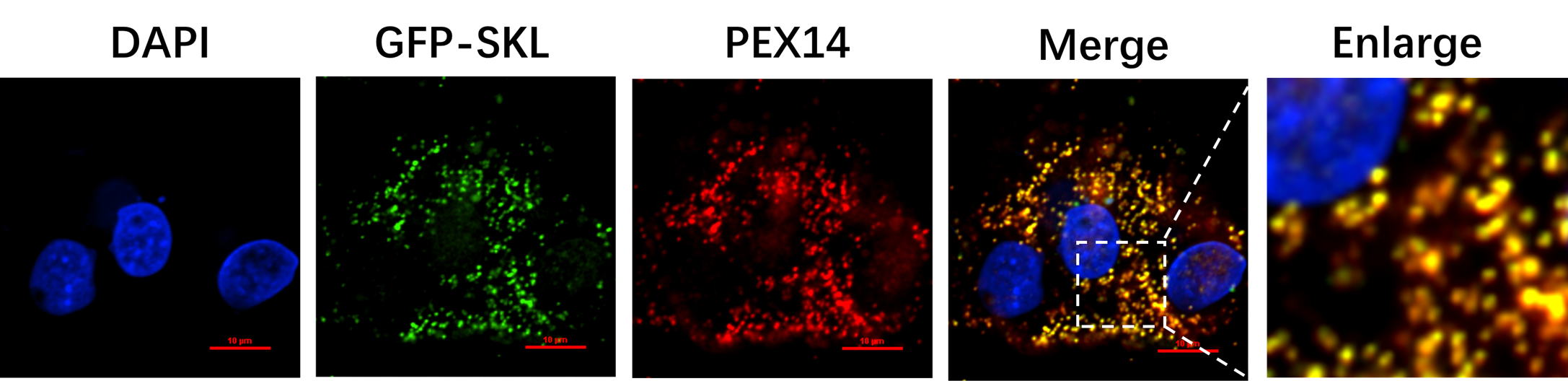

Supplement: S4 Fig — LLC-PK1 cells were transfected with the GFP-SKL plasmid and then cells were fixed for IFA using anti-PEX14 antibody. Nuclei were counterstained with DAPI. Scale bar, 10 µm. (TIF) [file ppat.1013163.s004.tif]

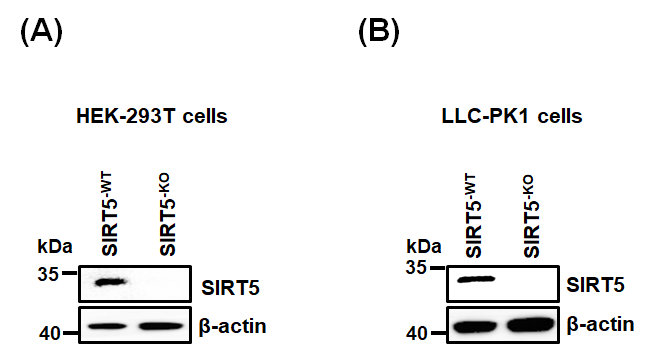

Supplement: S5 Fig — (A-B) Western blotting analysis to identify SIRT5 KO HEK-293T cells (A) and LLC-PK1 cells (B). (TIF) [file ppat.1013163.s005.tif]

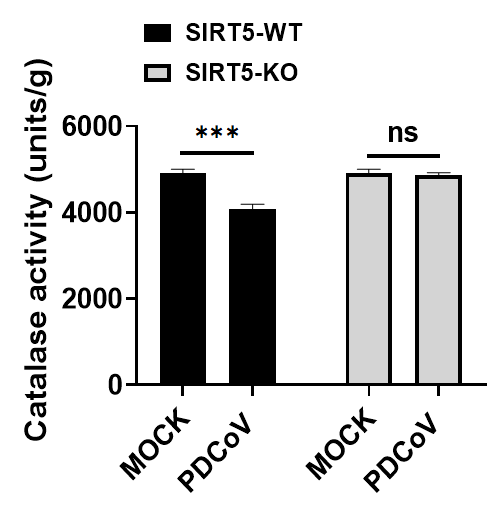

Supplement: S6 Fig — Catalase activity assay in WT and SIRT5-KO LLC-PK1 cells under mock or PDCoV-infected cells. (TIF) [file ppat.1013163.s006.tif]

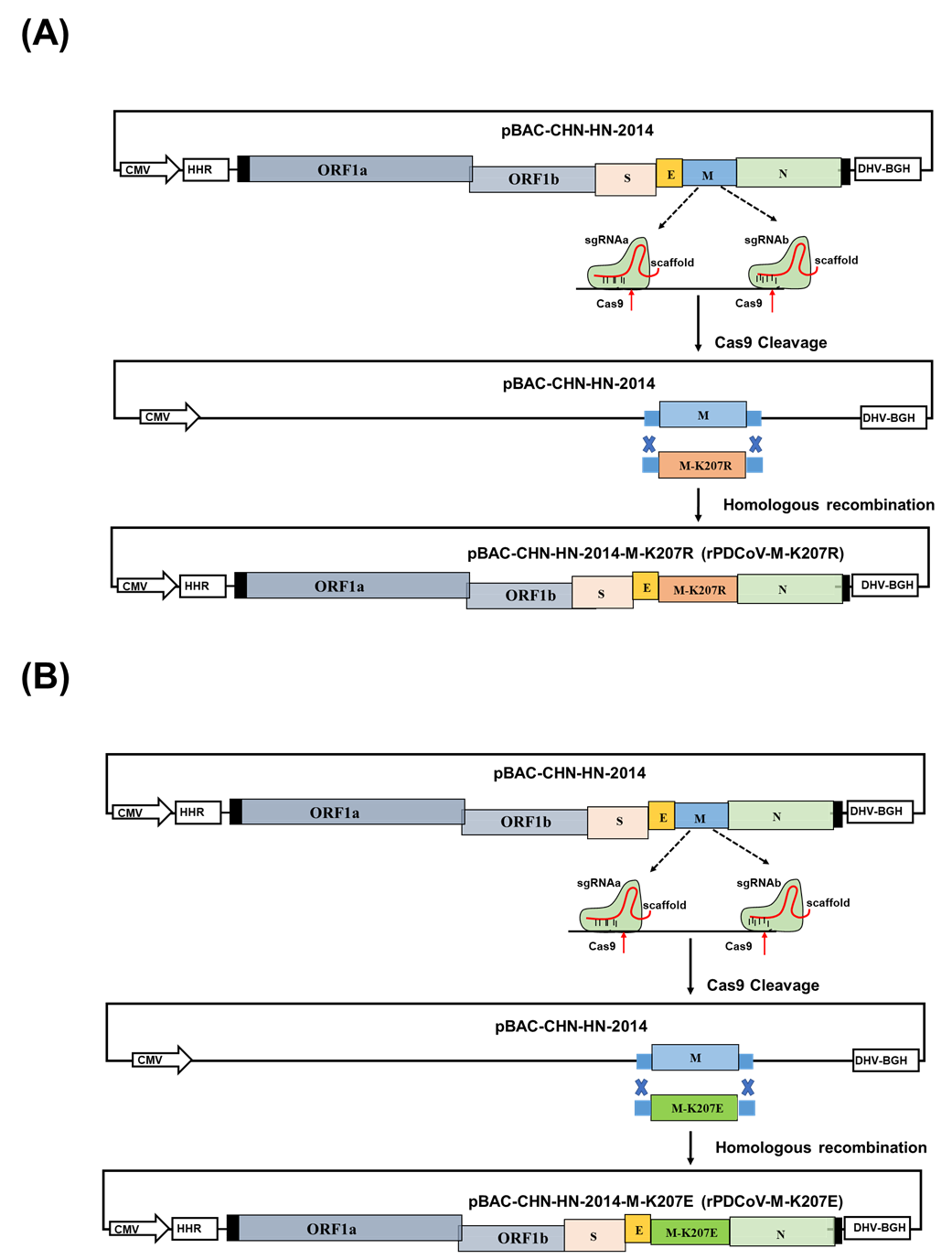

Supplement: S7 Fig — (A–B) Recombinant PDCoV with K207R or K207E mutations in the M gene was generated using CRISPR/Cas9. The sgRNAs targeting sequences flanking the mutation site facilitated Cas9-mediated linearization of the pBAC-CHN-HN-2014 plasmid. Mutant M gene fragments were created via overlapping PCR and inserted into the vector through homologous recombination, yielding recombinant plasmids pBAC-CHN-HN-2014-M-K207R (A) and -K207E (B). Recombinant plasmids were transfected into LLC-PK1 cells, generating recombinant viruses rPDCoV-M-K207R and rPDCoV-M-K207E, respectively. (TIF) [file ppat.1013163.s007.tif]

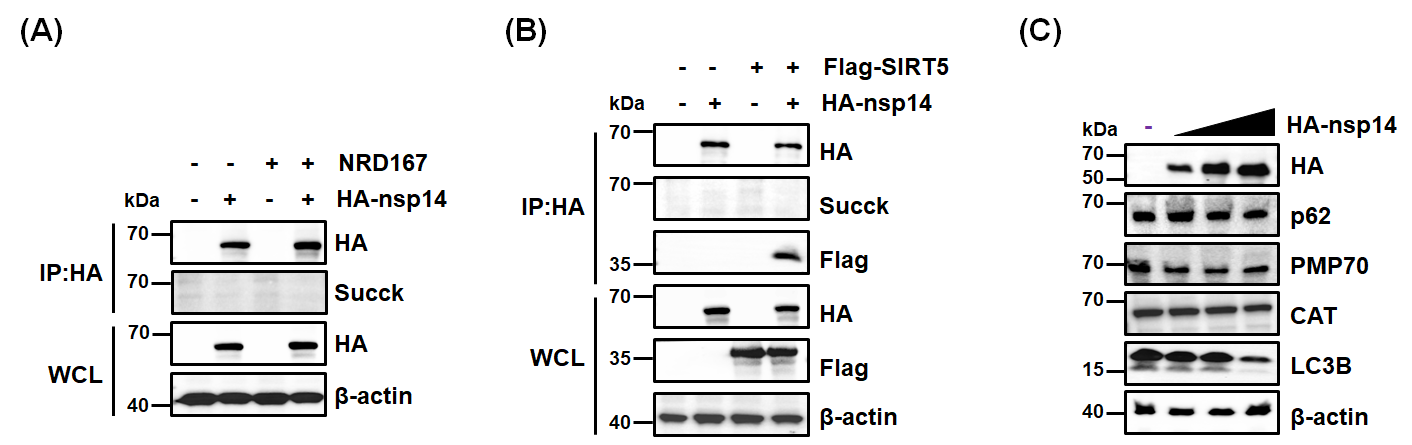

Supplement: S8 Fig — (A) HEK-293T cells were transfected with pCAGGS-HA-nsp14 or empty vector, followed by treatment with NRD167 (10 µM). The cells were lysed, and immunoprecipitation was performed using anti-HA antibody. WCL and IP complexes were analyzed by western blotting. (B) HEK-293T cells were co-transfected with pCAGGS-HA-nsp14 and pCAGGS-Flag-SIRT5, along with empty vector controls. The cells lysates were subjected to co-immunoprecipitation assay with anti-HA antibody and subsequent western blotting. (C) HEK-293T cells were transfected with pCAGGS-HA-nsp14 at increasing doses, then collected for western blotting analysis. (TIF) [file ppat.1013163.s008.tif]

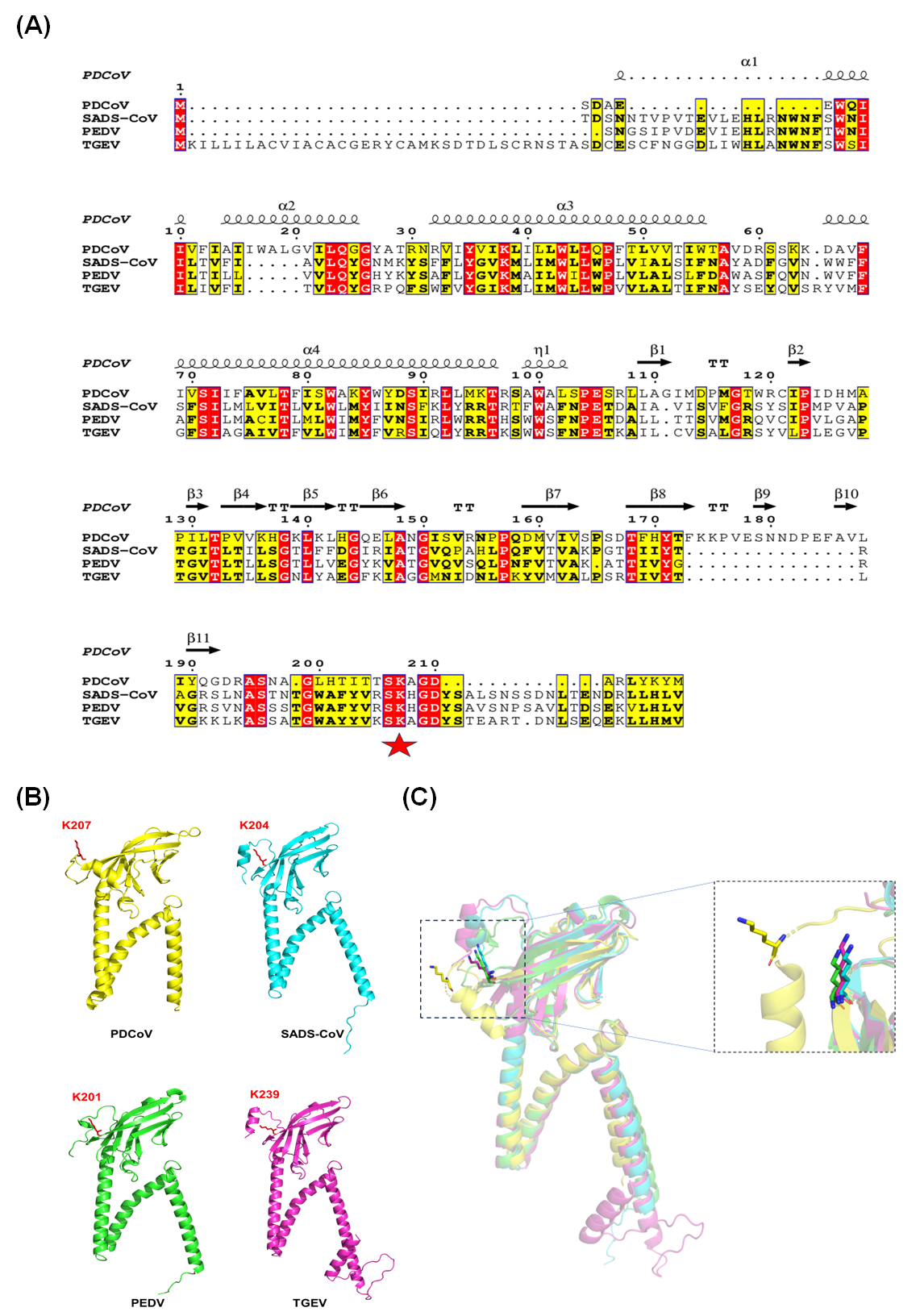

Supplement: S9 Fig — (A) Alignment of the amino acid sequences of the M proteins of PDCoV, SADS-CoV, PEDV and TGEV. The lysine 207 site of the porcine coronavirus M protein is indicated by a pentagram. (B-C) The three-dimensional structures of PDCoV M (yellow), SADS-CoV M (blue), PEDV M (green), and TGEV M (purple) obtained from Alpha Fold were analyzed with PyMOL software. (TIF) [file ppat.1013163.s009.tif]

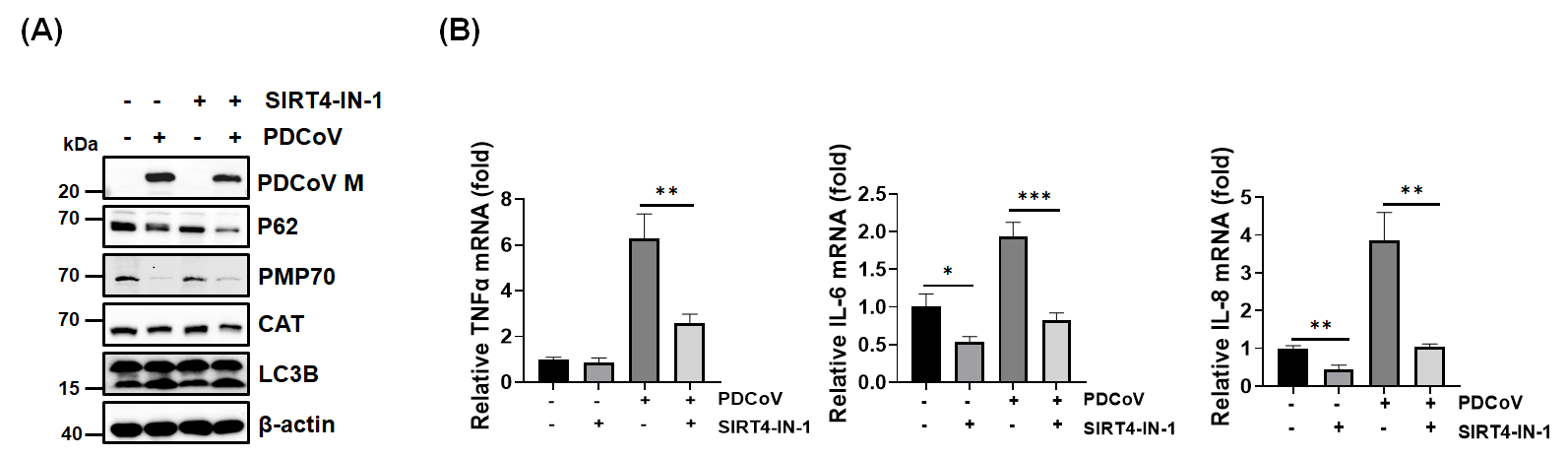

Supplement: S10 Fig — (A) LLC-PK1 cells were treated with SIRT4-IN-1 (100 µM) or left untreated, then infected with PDCoV for 12 h. Cells were harvested, and lysates were analyzed by western blotting with the indicated antibodies. (B) LLC-PK1 cells were treated with SIRT4-IN-1 (100 µM) or left untreated, then infected with PDCoV for 12 h. The cells were collected and subjected to RT-qPCR. (TIF) [file ppat.1013163.s010.tif]

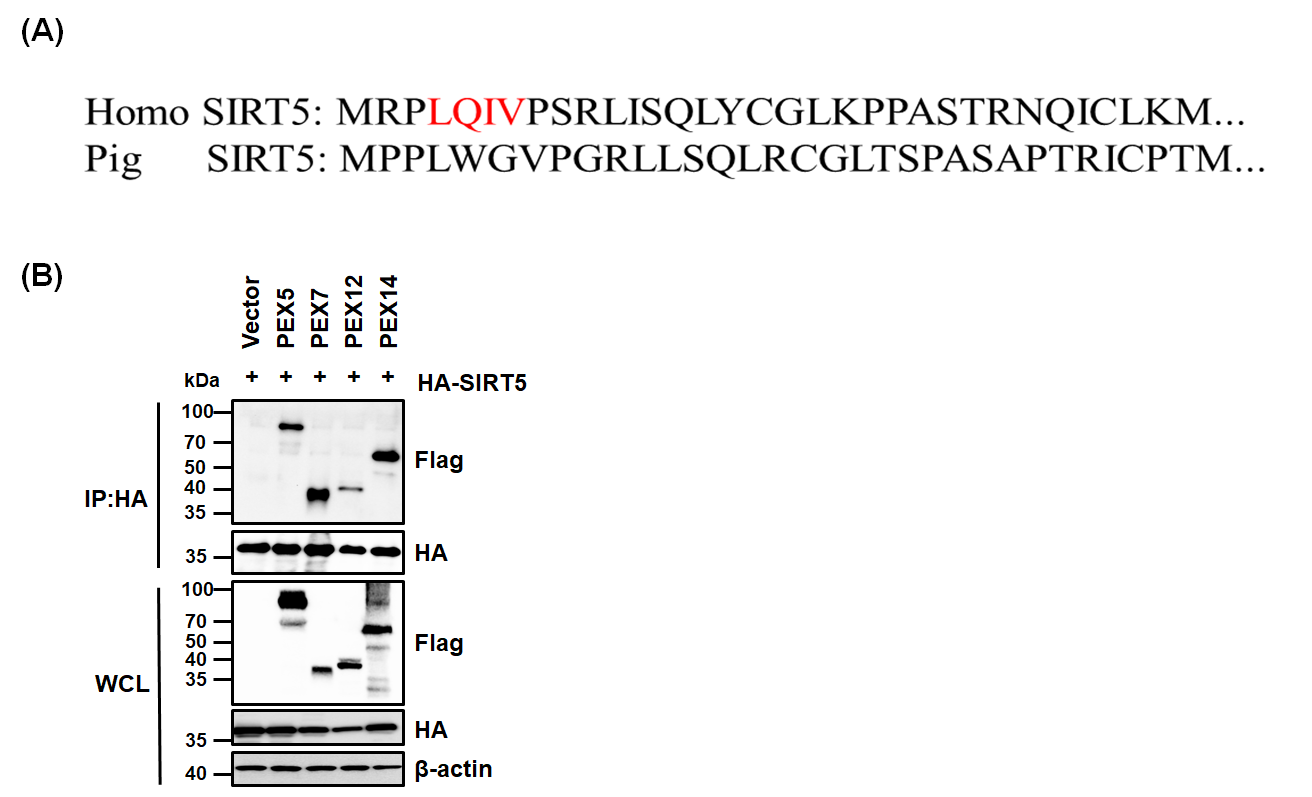

Supplement: S11 Fig — (A) Sequence alignment of N-terminal regions of porcine and human SIRT5, highlighting the divergent residues. (B) HEK-293T cells were co-transfected with pCAGGS-HA-SIRT5 and expression constructs encoding Flag-PEX5, PEX7, PEX12, or PEX14 for 24 h. Cell lysates were subjected to co-immunoprecipitation with anti-HA antibody, followed by western blotting analysis. (TIF) [file ppat.1013163.s011.tif]

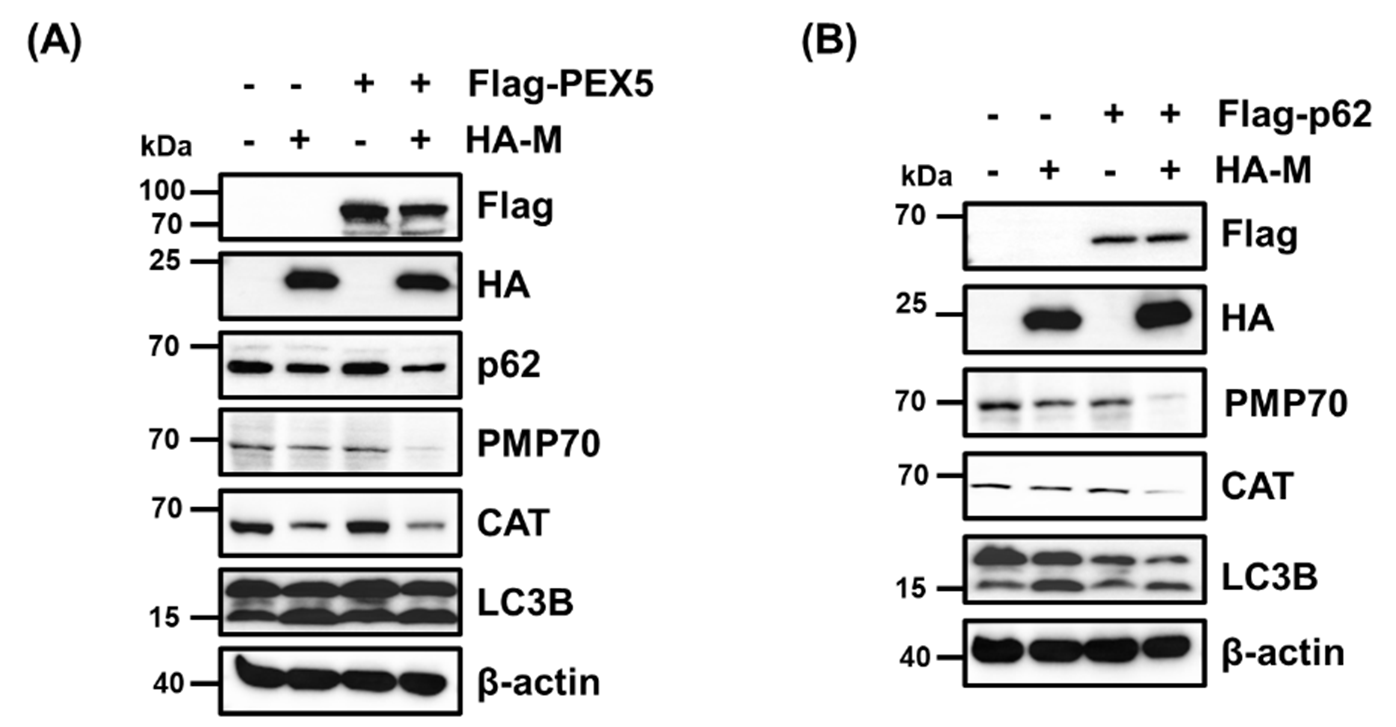

Supplement: S12 Fig — HEK-293T cells were co-transfected with pCAGGS-HA-M and either pCAGGS-Flag-PEX5 (A) or pCAGGS-Flag-p62 (B), along with empty vector controls. After 24 h, cell lysates were subjected to western blotting to detect the expression of HA-tagged M protein, Flag-tagged PEX5 or p62, and peroxisomal markers (PMP70, CAT), as well as LC3B as an autophagy marker. (TIF) [file ppat.1013163.s012.tif]

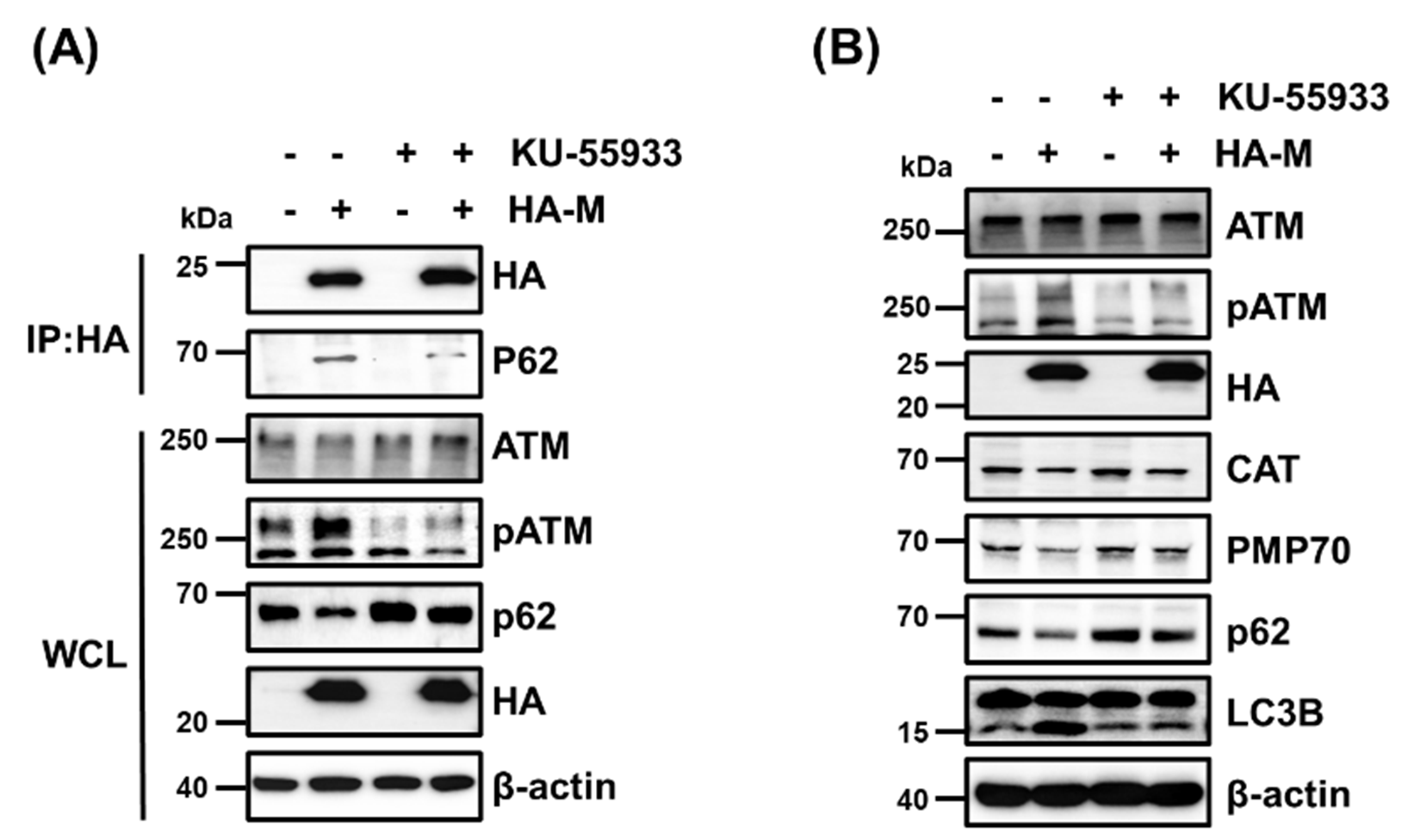

Supplement: S13 Fig — (A) HEK-293T cells were transfected with pCAGGS-HA-M or empty vector, followed by treatment with KU-55933 (10 µM). The cells lysates were subjected to co-immunoprecipitation assay with anti-HA antibody and subsequent western blotting. (B) HEK-293T cells were treated as in (A). Total cell lysates were analyzed by western blotting. (TIF) [file ppat.1013163.s013.tif]
